# Manipulation of prenylation reactions by structure-based engineering of bacterial indolactam prenyltransferases

**DOI:** 10.1038/ncomms10849

**Published:** 2016-03-08

**Authors:** Takahiro Mori, Lihan Zhang, Takayoshi Awakawa, Shotaro Hoshino, Masahiro Okada, Hiroyuki Morita, Ikuro Abe

**Affiliations:** 1 Graduate School of Pharmaceutical Sciences, The University of Tokyo, 7-3-1 Hongo, Bunkyo-ku, Tokyo 113-0033, Japan; 2 Department of Medicinal Resources, Institute of Natural Medicine, University of Toyama, 2630-Sugitani, Toyama 930-0194, Japan

## Abstract

Prenylation reactions play crucial roles in controlling the activities of biomolecules. Bacterial prenyltransferases, TleC from *Streptomyces blastmyceticus* and MpnD from *Marinactinospora thermotolerans*, catalyse the ‘reverse' prenylation of (−)-indolactam V at the C-7 position of the indole ring with geranyl pyrophosphate or dimethylallyl pyrophosphate, to produce lyngbyatoxin or pendolmycin, respectively. Using *in vitro* analyses, here we show that both TleC and MpnD exhibit relaxed substrate specificities and accept various chain lengths (C_5_–C_25_) of the prenyl donors. Comparisons of the crystal structures and their ternary complexes with (−)-indolactam V and dimethylallyl *S*-thiophosphate revealed the intimate structural details of the enzyme-catalysed ‘reverse' prenylation reactions and identified the active-site residues governing the selection of the substrates. Furthermore, structure-based enzyme engineering successfully altered the preference for the prenyl chain length of the substrates, as well as the regio- and stereo-selectivities of the prenylation reactions, to produce a series of unnatural novel indolactams.

## Abstract (web summary)

Regioselective prenylation of small aromatic natural molecules is crucial for their biological activity. Here, the authors present the biochemical and structural characterisation of two prenyltransferases and a structure-based engineering strategy to modulate their substrate specificity.

## Graphical abstract

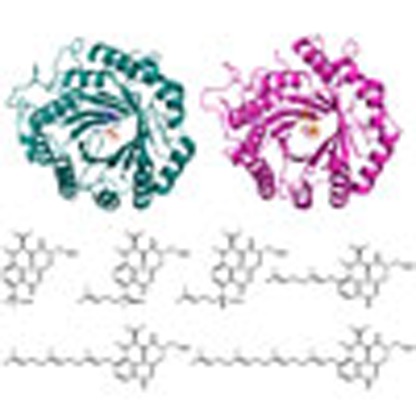

As prenylated molecules usually exhibit improved interactions with proteins and biological membranes, prenylation reactions catalysed by prenyltransferases (PTs) play crucial roles in controlling the activities of biomolecules, as exemplified by the biosyntheses of Vitamin K and menaquinones in primary metabolism^1^, the posttranslational modifications of peptides^2^,^3^ and proteins^4^, as well as the production of various biologically active secondary metabolites in plants, fungi and bacteria^5^,^6^,^7^,^8^,^9^. This is also the case for the indole alkaloids, which include the specific cell cycle inhibitors tryprostatins^5^, the ergot alkaloids anti-migraine drugs^6^ and the potent protein kinase C activator teleocidins^7^ (Fig. 1). In their biosyntheses, soluble indole PT superfamily enzymes, which are the most common aromatic PTs in bacteria and fungi, usually select the dimethylallyl pyrophosphate (DMAPP) as the prenyl donor to catalyse the Friedel–Crafts alkylation of the indole ring, thereby contributing to the structural diversification and exertion of their prominent biological activities^10^,^11^,^12^,^13^. In nature, enzymes that regioselectively catalyse the prenylation of all of the possible positions of the indole ring have been identified^10^,^11^,^12^,^13^ (Supplementary Figs 1 and 2). There are two types of the prenyltransfer reactions: ‘normal' prenylation (attack at the C-1 primary centre of the prenyl carbocation) and ‘reverse' prenylation (attack at the C-3 tertiary centre of the prenyl carbocation). These indole PT enzymes share the αββα (ABBA) barrel overall structural fold; however, the structure–function relationships of the enzymes still remained to be elucidated and the structure-based rational control of the enzyme reactions has been awaited.

We recently reported the biosynthetic gene cluster for teleocidin B, a unique prenylated indolactam with a monoterpenoid moiety fused with C-6 and C-7 of the indole ring^14^ (Fig. 1a). The gene cluster from *S. blastmyceticus* encodes an indolactam PT, TleC, which is a 42-kDa protein consisting of 391 amino acids that shares 40% and 38% identities with LtxC from the cyanobacterium *Moorea producens*^15^ and MpnD from the deep sea bacterium *M. thermotolerans*^16^, respectively (Supplementary Fig. 3). Heterologous expression in *Streptomyces lividans* TK21 and an *in vivo* analysis revealed that TleC catalyses the ‘reverse' prenylation of the cyclic dipeptide (−)-indolactam V (**1**) at the C-7 position of the indole ring with geranyl pyrophosphate (GPP) (C_10_) to produce lyngbyatoxin A (**2**), with the C19*R* configuration, as in the case of LtxC^15^ (Fig. 1a). MpnD also catalyses the ‘reverse' prenylation of **1** at the C-7 position, with DMAPP (C_5_) to yield pendolmycin^16^ (**3**) (Fig. 1b). The two bacterial PTs thus yield products with different prenyl chain lengths from the common indolactam. Furthermore, notably, the ‘reverse' prenylation at the C-7 position of the indole ring is limited only to the enzyme reactions catalysed by TleC, LtxC and MpnD, although several ‘reverse' prenylation enzymes have been reported^17^,^18^,^19^.

Here we report the biochemical characterization, crystal structures and structure-based engineering of *S. blastmyceticus* TleC and *M. thermotolerans* MpnD. Interestingly, the indolactam-specific TleC and MpnD both accepted different chain lengths (from C_5_ to C_25_) of prenyl pyrophosphate *in vitro*. Comparisons of the crystal structures revealed the intimate structural details of the enzyme-catalysed reverse prenylation reactions. Furthermore, we succeeded in structure-based enzyme engineering, to rationally control the preference for the chain lengths of the prenyl donors, as well as the regio- and stereo-selectivities of the enzyme reactions, to generate unnatural novel indolactams.

## Results

### *In vitro* characterizations of TleC and MpnD

*S. blastmyceticus* TleC and *M. thermotolerans* MpnD were heterologously expressed in *Escherichia coli* as His-tagged fusion proteins and purified to homogeneity by Ni-chelate affinity chromatography. Although most of the aromatic PTs and soluble indole PTs are metal ion-independent enzymes, divalent cations such as Mg^2+^, Ca^2+^ and Co^2+^ are known to affect the enzyme activities^10^,^11^,^12^,^13^. Notably, a recent study on an insect isoprenyl diphosphate synthase demonstrated that the prenyl chain length of the enzyme reaction products is controlled by metal ions^20^. Therefore, we first investigated the metal ion dependency of the enzyme reactions, by using (−)-indolactam V and GPP/DMAPP as substrates in the presence of various metal ions (Mg^2+^, Ca^2+^, Co^2+^, Ni^2+^, Mn^2+^, Fe^2+^, Zn^2+^ or Cu^2+^). The results demonstrated that the presence of metal ions did not change the product profile of the enzyme reactions but the presence of Mg^2+^ and Ca^2+^ slightly enhanced (<10%) their activities. Furthermore, the enzyme activities were not influenced by the addition of the chelating agent EDTA (Supplementary Fig. 4). These results clearly indicated that both TleC and MpnD are also metal ion-independent enzymes, with activities slightly enhanced by the addition of Mg^2+^ and Ca^2+^, as in the cases of other indole PTs^10^,^11^,^12^,^13^.

**Figure 2:**
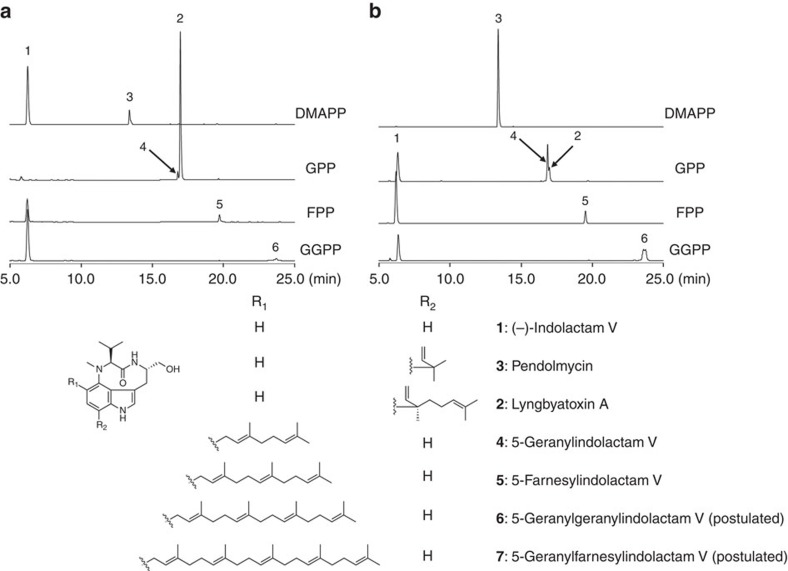
The *in vitro* enzyme reactions of *S. blastmyceticus* TleC and *M. thermotolerans* MpnD. HPLC elution profiles of the enzyme reaction products of (**a**) TleC and (**b**) MpnD from (−)-indolactam V and prenyl pyrophosphates with various chain lengths as substrates.

Next, to investigate the substrate specificities of TleC and MpnD, the purified recombinant enzymes were incubated with several indole derivatives and prenyl pyrophosphates with various chain lengths as substrates (Supplementary Fig. 5). The liquid chromatography–mass spectrometry (LC–MS) analysis of the enzyme reaction products demonstrated that both enzymes exhibit strict substrate specificity towards the prenyl acceptors. Thus, they are indolactam-specific and do not accept indole, 4-aminoindole, 4-methoxyindole, indole acetic acid, L-tryptophan, harman and brevianamide F as a prenyl acceptor. In contrast, both TleC and MpnD exhibited relaxed substrate specificities towards the prenyl donors. First, the indolactam-specific TleC and MpnD both accepted DMAPP (C_5_) and GPP (C_10_) as substrates to produce pendolmycin (**3**), and lyngbyatoxin A (**2**) and 5-geranylindolactam V (**4**) (**2**:**4**=26:1 for TleC; **2**:**4**=1:3 for MpnD), respectively (Fig. 2). The structure of **3** and **2** were determined by comparisons of the NMR data with the reported data. TleC exhibited 23-fold higher *k*_cat_/*K*_M_ value for the C_10_ prenylation activity over the C_5_ prenylation activity. In contrast, MpnD showed 109-fold higher activity for the C_5_ over the C_10_ (Table 1). Thus, TleC and MpnD clearly prefer to accept GPP and DMAPP as the prenyl donors, respectively. Meanwhile, the high-resolution-mass spectrometry (HR-MS) of **4** revealed a 136-Da larger molecular weight than that of **1**, suggesting the presence of a geranyl group in compound **4**. The ^1^H-NMR signals at *δ*_H_ 5.15 (1H, *t*, *J*=6.8 Hz) and 5.30 (1H, *t*, *J*=6.4 Hz) of the geranyl moiety and *δ*_H_ 7.04 (1H, *d*, *J*=2.0 Hz), 7.12 (1H, *d*, *J*=8.0 Hz), 7.24 (1H, *d*, *J*=8.0 Hz) and 8.26 (1H, *brs*) of the indole ring indicated that GPP is attached to the C-5 or C-7 position of the indolactam in the ‘normal' manner (the key signals due to the methylidene group in the ‘reverse' prenylated structure are missing). Further ^1^H-detected multi-bond heteronuclear multiple quantum coherence spectra revealed that H-19a and H-19b (*δ*_H_ 3.73 and *δ* 3.55, 1H, *dd*, *J*=6.5 Hz) correlated with C-4 (*δ*_c_ 141.8), indicating prenylation at the C-5 position (Supplementary Fig. 6a). The structure of **4** was thus determined to be 5-geranylindolactam V (Fig. 2).

The relaxed substrate specificity of the enzymes prompted us to test longer chain prenyl donors as substrates. Interestingly, both TleC and MpnD also accepted farnesyl pyrophosphate (FPP) (C_15_), geranylgeranyl pyrophosphate (GGPP) (C_20_) and geranylfarnesyl pyrophosphate (GFPP) (C_25_) as prenyl donors, to produce **5**, **6** and **7**, respectively (Fig. 2 and Supplementary Fig. 7). HR-MS indicated that **5** was C_32_H_46_N_3_O_2_, corresponding to the farnesylated **1**. The ultraviolet and NMR spectra of **5** were quite similar to those of **4**, except for the presence of an additional isoprenyl unit (*δ*_H_ 1.59, 1.97, 2.06, and 5.09; *δ*_C_ 17.7, 26.8, 39.8, 124.4, and 131.3) (Supplementary Figs 6b and 8). Thus, the structure of **5** was determined to be 5-farnesylindolactam V. In a similar manner, **6** and **7** are postulated to be 5-geranylgeranylindolactam V and 5-geranylfarnesylindolactam V, respectively (Fig. 2). In contrast to typical indole PTs that exhibit broad substrate specificities towards the prenyl acceptors but with strict specificities towards the prenyl donors^10^,^11^,^12^,^13^, it is remarkable that the indolactam-specific TleC and MpnD exhibited unusually relaxed substrate specificities towards the prenyl donors and accepted different chain lengths (from C_5_ to C_25_) of prenyl pyrophosphate *in vitro*. Although TleC and MpnD predominantly catalyse the ‘reverse' prenylation of (−)-indolactam V at the C-7 position of the indole ring with GPP (C_10_) or DMAPP (C_5_), respectively, these enzymes also catalysed the C-5 ‘normal' prenylation *in vitro* when longer chain prenyl donors (C_15_, C_20_ and C_25_ in TleC and C_10_, C_15_, C_20_ and C_25_ in MpnD) were used as substrates. These results suggested that the binding modes of the longer chain substrates are different from those of the physiological substrates. An indole PT BAE61387 from *Aspergillus oryzae* DSM1147 reportedly catalyses the prenylation of up to the C_15_ FPP^21^. However, to the best our knowledge, this is the first demonstration that indole PT enzymes catalyse the C_20_ and C_25_ prenylation reactions.

### Overall structures of TleC and MpnD

To understand the intimate structural details of the enzyme reactions, we solved the X-ray crystal structures of TleC and MpnD as the apo structures (at 2.0 and 1.6 Å resolutions, respectively) and the ternary complexes with (−)-indolactam V and a DMAPP analogue dimethylallyl *S*-thiophosphate (DMSPP) (at 2.1 and 1.4 Å resolutions, respectively) (Table 2). The overall structures of TleC and MpnD adopt the ABBA-fold, which consists of ten antiparallel β-strands assembled into a circular β-barrel, surrounded by a ring of solvent-exposed α-helices, as observed in the structures of indole PTs and aromatic PTs^22^,^23^,^24^,^25^,^26^,^27^ (Supplementary Fig. 9). Furthermore, the overall structures of TleC and MpnD are highly homologous to each other, with a root mean square deviation (RMSD) of 1.9 Å for the Cα-atoms. The structural comparison using the Dali programme revealed that the TleC structure shares high similarities with those of the known indole PTs, including *Neosartorya fischeri* AnaPT^24^, *Aspergillus fumigatus* FgaPT2 (ref. 25) and *A. fumigatus* FtmPT1 (ref. 26), with RMSD values of 2.7, 2.8 and 2.8 Å for the Cα-atoms, respectively. MpnD is also quite similar to these enzymes, with RMSD values of 2.9, 3.0 and 2.8 Å, respectively. A structure-based sequence comparison indicated that TleC and MpnD share low sequence identity/similarity to FgaPT2 (TleC: 14/27%, MpnD: 12/24%), FtmPT1 (TleC: 12/24%, MpnD: 15/25%), CdpNPT (TleC: 12/25%, MpnD: 13/25%) and AnaPT (TleC: 16/29%, MpnD: 15/26%) (Supplementary Fig. 10). The comparison of overall structures of TleC, MpnD and the structurally characterized other indole PTs revealed that the indole PT's conserved amino-terminal α-helices, locating outside of β-barrel, are converted into loops and cover the centre of circular β-barrel in TleC and MpnD. Furthermore, TleC and MpnD also lack the two helices between α4 and β3, and β4 and α5, corresponding to α6 and α7 in the other indole PTs.

### Substrate binding

**Figure 3:**
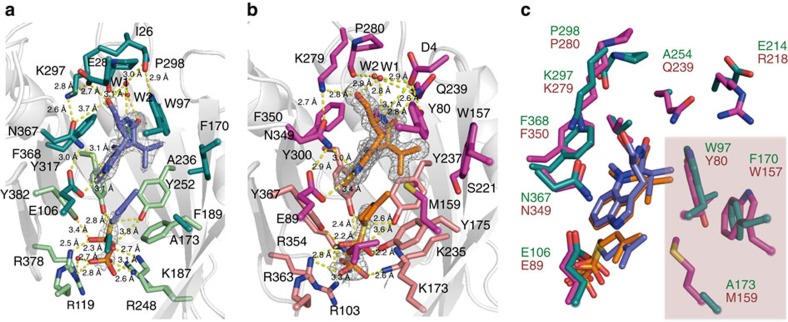
Comparison of the substrate binding modes in *S. blastmyceticus* TleC and *M. thermotolerans* MpnD. Closeup views of the active site architectures in (**a**) the TleC-**1**-DMSPP ternary complex and (**b**) the MpnD-**1**-DMSPP ternary complex. (**c**) Superimposition of the active site residues of TleC and MpnD. The key three residues discussed here are highlighted by red squares. (−)-Indolactam V (**1**) and DMSPP are depicted by blue and orange stick models, respectively. The amino acid residues located in the pyrophosphate and indolactam-binding sites are represented by limegreen and green stick models in TleC, and salmon and magenta stick models in MpnD, respectively. The *F*_o_−*F*_c_ electron density maps of the **1** and DMSPP are represented as a black mesh, contoured at +2.5*σ*. Dashed yellow lines represent hydrogen bonds. Red spheres depict the water molecules.

The ternary complex structures revealed the binding modes of their substrates. TleC binds the (−)-indolactam V (**1**) and DMSPP in the centre of the β-barrel and the catalytic Glu106 protrudes towards the N-1 of the indole ring, as in the cases of other indole PTs^24^,^25^,^26^,^27^ (Fig. 3a and Supplementary Figs 11a and 12a). A hydrogen bond network is formed among Glu28, Glu106, Lys297 and Asn367 neighbouring **1**, in which Glu106 and Asn367 hydrogen bond with the N-1 of **1**, while Lys297 and Glu28 hydrogen bond with the oxygen atom of the 14-OH of **1**. Furthermore, **1** is also fixed by hydrogen bond networks among the C-11 carbonyl oxygen, the N-10 of **1**, the main chain amine of Ile26, the N-1 of Trp97 and the main chain carbonyl of Pro298 via two water molecules W1 and W2. In contrast, the α and β-phosphates of DMSPP are anchored by positively charged residues, Arg119, Lys187, Arg248 and Arg378. In addition, the α-phosphate is held by the so-called ‘tyrosine shield' composed of Tyr252, Tyr317 and Tyr382. These tyrosines have been proposed to play an important role in stabilizing the dimethylallyl cation during the enzyme reaction^24^,^25^,^26^,^27^. Finally, the dimethylallyl moiety is sandwiched between the indole ring of **1** and the benzene ring of Phe189, and is stabilized by *π*-*π* interactions.

**Figure 4:**
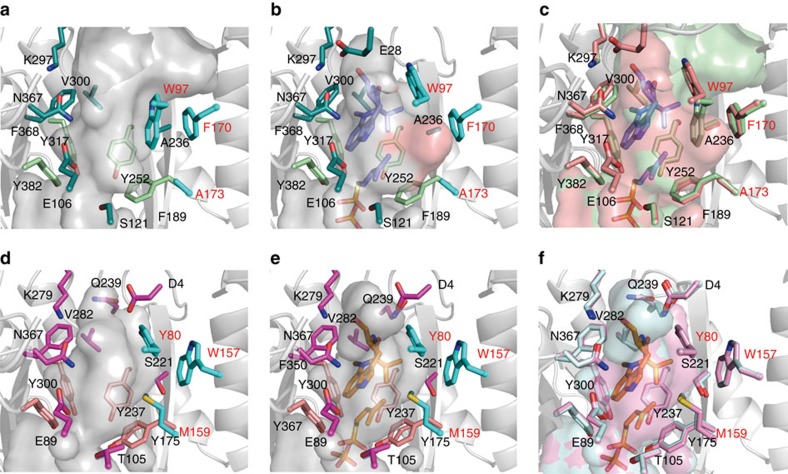
Comparison of the active site structures of *S. blastmyceticus* TleC and *M. thermotolerans* MpnD. Closeup views of the active site cavities of (**a**) the TleC apo structure and (**b**) the TleC complex structure with **1** and DMSPP, (**c**) superimposed view of apo and complex structure of TleC, (**d**) the MpnD apo structure, (**e**) the MpnD complex structure with **1** and DMSPP, and (**f**) superimposed view of apo and complex structure of MpnD. (−)-Indolactam V (**1**) and DMSPP are depicted by a cyan and blue stick models, respectively. The amino acid residues located in the pyrophosphate and indolactam-binding sites are represented by limegreen and green stick models in TleC, and magenta and salmon stick models in MpnD, respectively. The three important residues discussed in the main text are depicted by cyan stick models. The ‘prenyl binding pocket' in the TleC-**1**-DMSPP ternary complex structure is highlighted by a red surface. The residues and surface of apo structure and complex structure of TleC are represented by palegreen and salmon, respectively. The residues and surface of apo structure and complex structure of MpnD are represented by pink and pale cyan, respectively.

The active site architecture of the ternary structure revealed that MpnD binds **1** and DMSPP in a similar manner to that of TleC, even though these enzymes share only moderate amino acid identities (Fig. 3b and Supplementary Figs 11b and 12b). In addition to Glu89, Lys279, Pro280, Asn349 and the two structurally conserved water molecules (corresponding to Glu106, Lys297, Pro298, Asn367, W1 and W2 in TleC, respectively), Asp4, Tyr80 and Gln239 participate in the tight hydrogen bond network with **1**. In MpnD, the two water molecules, along with Asp4, Tyr80 and Gln239, form further hydrogen bonds with the N-10, C-11 carbonyl and 14-OH of **1**, to tightly accommodate the indolactam. In contrast, in the previously reported crystal structures of indole PTs, which exhibited relatively broad substrate specificity for prenyl acceptors, the indole substrate is only fixed by a few hydrogen bonds, in addition to a hydrogen bond with highly conserved glutamic acid, while the pyrophosphate is anchored tightly by numerous salt bridges, electrostatic interactions and hydrogen bond networks^25^,^27^. For example, the structure of CdpNPT complexed with (*S*)-benzodiazepinedione revealed that only hydroxyl group of Thr108 construct a hydrogen bond with O-13′ (ref. 27). Furthermore, the structure of FgaPT2 complexed with substrate L-tryptophan also indicated that the indole substrate is only fixed by hydrogen bonds with side chain of Arg244, main chain of Ile80 and Leu81 (ref. 25). Thus, the presence of the tight hydrogen bond networks in TleC and MpnD would explain why they are both indolactam specific and do not accept other indole derivatives as the prenyl acceptor substrate. In contrast, as in the case of TleC, the dimethylallyl moiety is sandwiched between the indole ring of **1** and the benzene ring of Tyr175 in MpnD. The binding mode of DMAPP is thus quite similar; however, the amino acid residues lining the prenyl binding pocket Trp97, Phe170 and Ala173 in TleC are uniquely substituted with Tyr80, Trp157 and Met159, respectively, in MpnD (Figs 3c and 4, and Supplementary Figs 11–13). Notably, Trp97 in TleC and the corresponding Tyr80 in MpnD are located on the so-called ‘substrate-binding loop' between α2 and β1, which was proposed to be important for substrate binding by forming a hydrogen bond network between the substrate and the backbone oxygens of Leu94 and Met94 in FgaPT2 and FtmPT1, respectively^25^,^26^.

Remarkably, a comparison of the active site architectures of the TleC apo structure and the ternary complex structure with substrates revealed that the TleC-specific Trp97 rotated by about 70° when the substrates bound to the active site (Fig. 4a–c). This rotation generated a new large pocket to accommodate the prenyl side chain of the C_10_ GPP substrate in TleC, but this pocket is absent in the active site of MpnD. The pocket is mainly composed of hydrophobic residues Trp97, Glu123, Phe170, Ala173 and Phe189 at the superior region of the isoprenyl moiety of DMSPP. In contrast, the rotation of the corresponding Tyr80 was not observed in the MpnD structures, because of the steric hindrance between Tyr80 and Met159 (Fig. 4d–f). This is due to the small-to-large substitutions of Phe170 and Ala173 in TleC with Trp157 and Met159 in MpnD and the different orientation of the side chain of Tyr80, corresponding to Trp97 in TleC, which leans slightly towards Met159 to form a hydrogen bond network with the carboxyl group of Asp4. Moreover, the significant conformational differences of the loop between residues 166–174 in TleC and 152–160 in MpnD (RMSD of 1.9 Å for the Cα atoms), and the lack of a residue between 157 and 159 in MpnD (170 and 173 in TleC), result in the displacement of the Cα atom of the active site forming residue Met159 in MpnD by 1.3 Å towards the inside of the active site, as compared with Ala173 in TleC. These observations along with the similar binding modes of substrates between TleC and MpnD suggested that the presence of the ‘prenyl binding pocket' controls the preference of the chain length of the prenyl donors, as well as the regio- and stereo-selectivities of the enzyme-catalysed prenylation reactions (Fig. 3c).

### Structure-based engineering of TleC and MpnD

**Figure 5:**
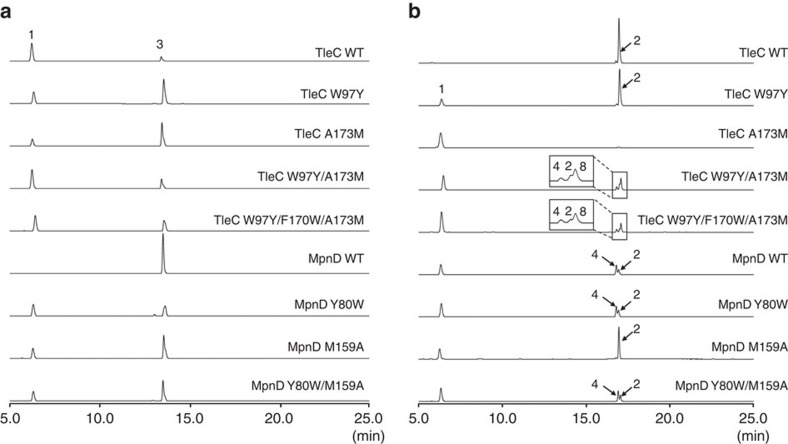
The *in vitro* enzyme reactions of the wild-type and mutants of *S. blastmyceticus* TleC and *M. thermotolerans* MpnD. HPLC elution profiles of the enzyme reaction products from (−)-indolactam V and (**a**) DMAPP or (**b**) GPP as substrates.

On the basis of the structural analyses, we envisaged that the modulation of the ‘prenyl binding pocket' should alter the substrate and product specificities of the enzyme reactions. We therefore focused on the three uniquely substituted key residues lining the pocket: Trp97, Phe170 and Ala173 in *S. blastmyceticus* TleC and the corresponding Tyr80, Trp157 and Met159 in *M. thermotolerans* MpnD. To evaluate the importance of these residues, we first constructed the W97Y and A173M mutants for TleC and the Y80W and M159A mutants for MpnD, and investigated the effects of the mutagenesis on the enzyme activities with (−)-indolactam V and prenyl pyrophosphate (DMAPP, GPP or FPP) as substrates. Indeed, as anticipated, both the W97Y and A173M mutants of TleC significantly increased the activity for the C_5_ DMAPP substrate, to produce pendolmycin (**3**) (Fig. 5 and Table 1). Furthermore, remarkably, the A173M mutant no longer accepted the C_10_ GPP to yield lyngbyatoxin A (**2**). The kinetic analyses revealed that the TleC A173M mutant exhibits a 2.3-fold increase in the *k*_cat_/*K*_M_ value for the C_5_ prenylation activity, whereas the C_10_ prenylation activity was almost abolished, as compared with the wild-type enzyme. Thus, TleC was functionally converted to the C_5_ PT by the small-to-large A173M single amino acid substitution. In contrast, notably, the TleC W97Y mutant exhibited a 31-fold increase for the C_5_ prenylation activity, although it still retained the C_10_ prenylation activity comparable to that of the wild-type enzyme.

The Y80W and M159A mutants of *M. thermotolerans* MpnD both decreased the activity for the C_5_ substrate, but instead increased the C_10_ prenylation activity (Fig. 5 and Table 1). Furthermore, the large-to-small M159A substitution even increased the C_15_ prenylation activity. The kinetic analyses revealed that the M159A mutant exhibited an 8.8-fold decrease in the *k*_cat_/*K*_M_ value for the C_5_ prenylation activity, whereas it exhibited 4.7- and 3.8-fold increases in the *k*_cat_/*K*_M_ for the C_10_ and C_15_ prenylation activities, respectively. Moreover, surprisingly, the product ratio of lyngbyatoxin A (**2**) (the ‘reverse' C_10_ prenylation at the C-7 position of **1**) and 5-geranylindolactam V (**4**) (the ‘normal' C_10_ prenylation at the C-5 position of **1**) was significantly altered in the M159A mutant, which now produced **2** as the major product. In contrast, the Y80W mutant exhibited a 24-fold decrease in the C_5_ prenylation activity, but a 5.5-fold increase in the *k*_cat_/*K*_M_ value for the C_10_ activity and a 2.7-fold higher affinity to GPP. This would be due to the loss of the hydrogen bond network from the hydroxyl group of Tyr80 and the narrowed active site entrance. These results suggested that the hydrophobicity and the shape of the active site are important for the substrate binding.

To further modulate the ‘prenyl binding pocket', we constructed double and triple mutants of the three key residues: the W97Y/A173M and W97Y/F170W/A173M mutants of TleC and the Y80W/M159A and Y80W/W157F/M159A mutants of MpnD. As a result, although the double and triple mutants of TleC significantly decreased the activities by 33- and 28-fold, respectively, they still preferred to accept DMAPP as the prenyl donor (Fig. 5 and Table 1). Interestingly, a careful examination of the enzyme reaction products of these mutants revealed the newly generated novel product **8**, as the major product, in addition to **2** and **4** (**2**:**4**:**8**=1:0.6:2.3 for the W97Y/A173M mutant of TleC; **2**:**4**:**8**=1:1: 2.9 for the W97Y/F170W/A173M mutant of TleC) from GPP as the prenyl donor (Fig. 5 and Supplementary Fig. 14). The structure of **8** was established to be teleocidin A-2, which is the 19*S*-epimer of **2**, by comparisons of the NMR data. The ^1^H-NMR of **8** showed good accordance with **2**, except the signals due to the prenyl moiety (that is, slight differences in the chemical shifts of H-20, H-21 and H-23, which are completely identical to those of teleocidin A-2 in the literature^28^). Thus, the mutations affected the stereochemistry of the enzyme-catalysed prenylation reaction to produce 19*S*-**8** as the major product along with 19*R*-**2** and its regioisomer 5-gerany-**4**. In contrast, the MpnD double mutant exhibited C_10_ and C_15_ prenylation activities comparable to those of the wild-type enzyme, whereas the C_5_ activity was decreased by 11-fold. Unfortunately, as the MpnD triple mutant was expressed as an insoluble inclusion body, we could not evaluate its activities. These results clearly demonstrated that the structure-based site-directed mutagenesis successfully altered not only the preference for the chain length of the prenyl donors but also the regio- and stereo-selectivities of the enzyme reactions.

### Enzyme reaction mechanisms of TleC and MpnD

The mechanism for the formation of lyngbyatoxin A (**2**) was proposed to proceed through *N*-geranylindolactam V as an intermediate, suggesting the involvement of the initial ‘normal' prenylation at the N-1 position of (−)-indolactam V (**1**) and the subsequent aza-Claisen rearrangement in the installation of the geranyl moiety on the C-7 position of **1** (Supplementary Fig. 15)^29^. This hypothesis is based on the presence of blastmycetin E, which is easily converted into des-*O*-methyl olivoretin E, with the terpenoid moiety fused with C-6 and C-7 of the indole ring by the aza-Claisen rearrangement under mild acidic conditions. To test this hypothesis, we synthesized *N*-geranylindolactam V, according to the published method^30^, and incubated it with TleC. As a result, no conversion was observed after an 18-h incubation, even in the presence of methanol containing 1% acetic acid (Supplementary Fig. 16). This result suggested that *N*-geranylindolactam V is not the intermediate and the ‘reverse' prenylation at the C-7 position of the indolactam does not proceed through the aza-Claisen rearrangement. Furthermore, the crystal structures of TleC and MpnD complexed with the substrates suggested that the distance between the C-3 position of DMSPP and the C-7 position of **1** (3.3 Å in TleC and 3.1 Å in MpnD) is closer than that between the C-1 position of the prenyl substrates and the N-1 position of **1** (6.0 Å in TleC and MpnD) (Supplementary Fig. 17a). Moreover, the docking model of TleC with GPP also suggested that GPP binds in the ‘prenyl binding pocket‘ in the active site and the distances between the C-3 position of GPP and the C-7 position of **1**, and the C-1 position of the prenyl substrates and the N-1 position of **1**, are almost the same as those with DMSPP (3.3 and 5.8 Å, respectively) (Supplementary Fig. 17b). These results strongly suggested that both TleC and MpnD catalyse the ‘reverse' prenylation directly onto the C-7 position of **1** to produce **2** and **3**, respectively. Interestingly, the docking model of MpnD with GPP also suggested that the loss of the ‘prenyl binding pocket' causes the alteration of the GPP binding mode in the active site of MpnD. In this model, GPP is predicted to bind between **1** and the aromatic ring of Tyr175, and the distance between the C-5 of **1** and the C-1 of GPP (3.2 Å) is almost the same as that between the C-7 of **1** and the C-3 of GPP (3.1 Å) (Supplementary Fig. 17d). This would be the reason why MpnD produces not only the C-7 ‘reverse' prenylation product **2** but also the C-5 ‘normal' prenylation product **4**.

## Discussion

Our *in vitro* analyses of *S. blastmyceticus* TleC and *M. thermotolerans* MpnD revealed that these enzymes strictly recognize the cyclic dipeptide (−)-indolactam V as the prenyl acceptor. Remarkably, the indolactam-specific PTs accept different chain lengths of prenyl pyrophosphate, to generate a series of unnatural indolactam derivatives. Furthermore, although previous *in vivo* analyses of TleC and MpnD demonstrated that these enzyme catalyse C-7 ‘reverse' prenylation.

To the best our knowledge, this is the first demonstration that a single PT enzyme catalyses from the C_5_ to C_25_ prenylation reactions. In contrast, most of the soluble indole PTs from fungi and bacteria show broad substrate specificities towards the prenyl acceptors, but not for the prenyl donors, except for several recently reported indole PTs, including AnaPT from *N. fischeri*^31^, 6-DMAT_sa_ from *S. ambofaciens* and 6-DMAT_sv_ from *S. violaceusniger*, which accept both DMAPP and GPP as prenyl donors^32^, and BAE61387 from *A. oryzae* DSM1147, which accepts DMAPP, GPP and FPP as prenyl donors^21^. These characteristic features of TleC and MpnD make the enzymes excellent targets for the manipulation of the prenylation reactions.

The structure-based engineering of TleC and MpnD demonstrated that the modulation of the ‘prenyl binding pocket' successfully altered the substrate and product specificities of the enzyme reactions. In particular, the three uniquely substituted key residues lining the pocket, Trp97, Phe170 and Ala173 in *S. blastmyceticus* TleC, and the corresponding residues Tyr80, Trp157 and Met159 in *M. thermotolerans* MpnD, are important for determining the preference of the chain length of the prenyl donors, as well as the regio- and stereo-selectivities of the enzyme reactions. The large-to-small M159A substitution in MpnD newly create the ‘prenyl binding pocket', which is absent in the active site of the wild-type MpnD, to significantly enhance the C_10_ and C_15_ prenylation activities. In contrast, the small-to-large A173M substitution in the TleC mutant leads to the loss of the pocket, which results in a dramatic decrease in the C_10_ prenylation activity. Furthermore, the additional W97Y substitution in TleC decreases the steric hindrance between the enzyme and GPP, as the double and triple mutants were able to accept GPP as a prenyl substrate. However, the different configuration of the active site architecture results in the alteration of the binding mode of GPP. Although the C-3 methyl group of GPP should face towards Ser121 in the wild-type TleC, to produce **2** with the C19*R* configuration, the C-3 methyl group may turn in the opposite direction in the mutants, leading to the production of **8** with the C19*R* configuration (Supplementary Fig. 17). These results strongly support our proposal that both TleC and MpnD catalyse the ‘reverse' prenylation directly onto the C-7 position of **1**, to produce **2** and **3**, respectively.

**Figure 6:**
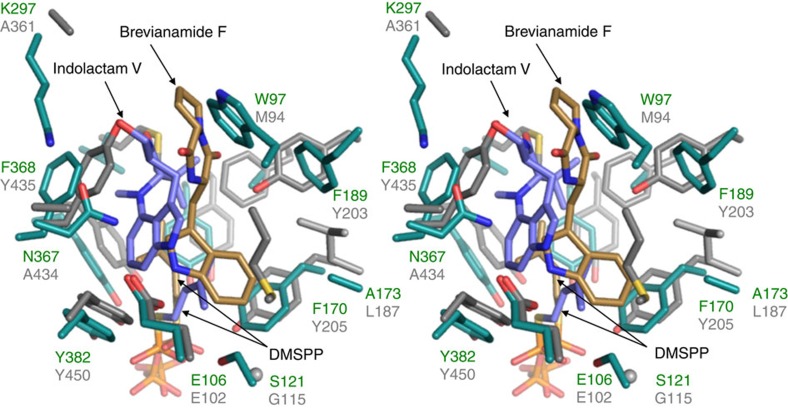
The stereo view of the active site structures of TleC and FtmPT1. The active site residues of TleC and FtmPT1 from *A. fumigatus* are shown by green and grey stick models, respectively. The respective substrates **1** and DMSPP in TleC, and brevianamide F and DMSPP in FtmPT1 are represented by blue and brown stick models, respectively. Structures were superposed based on the Cα-atom.

A comparison of the structures of the indolactam-specific TleC (and MpnD), with other indole PTs revealed that these enzymes share the ABBA barrel overall fold, but the modes of the substrate binding in the active site are totally different (Fig. 6 and Supplementary Fig. 18). The indole ring of the indolactam is positioned proximally to the side chains of Tyr317 and Tyr382 in TleC (Tyr300 and Tyr367 in MpnD) and the prenyl substrate is sandwiched between the indole ring and the benzene ring of Phe189, proximally to the side chain of Phe170 in TleC (Tyr175 and Met159 in MpnD, respectively). In contrast, in the crystal structures of FgaPT2 (ref. 25), FtmPT1 (ref. 26) and CdpNPT^27^ from *A. fumigatus*, their respective indole substrates, L-tryptophan, brevianamide F and (*S*)-benzodiazepinedione, bind in the opposite direction and are positioned proximally to the side chains of Lys174 and Tyr189 in FgaPT2, Leu187 and Tyr203 in FtmPT1 and in Val206 and Tyr221 in CdpNPT, respectively (the residues corresponding to Ala173 and Phe189 in TleC). In these cases, the dimethylallyl group of DMAPP is sandwiched by *π*–*π* interactions between the aromatic rings of the substrate and Tyr345 in FgaPT2 (Tyr382 in FtmPT1). Furthermore, Met328 and Tyr398 in FgaPT2 (Met364 and Tyr435 in FtmPT2, and Met349 and Trp419 in CdpNPT) protrude towards the active site centre to anchor the superior region of the dimethyl moiety of DMAPP (Supplementary Fig. 18d–f). In contrast, the corresponding residues are replaced with the slightly smaller amino acids Val and Trp in AnaPT^24^, Leu and Tyr in BAE61387 (ref. 21), and Val and Phe in TleC and MpnD, respectively. As a result, their active site pockets are relatively larger than those of the DMAPP-specific indole PTs (Supplementary Figs. 18a–c). Furthermore, the crystal structure of AnaPT and the model structure of LtxC from *M. producens*, which catalyses the same reaction as TleC, suggested that these enzymes also have the ‘prenyl binding pocket', and the three key residues discussed above are uniquely replaced with Gly102, Phe212 and Val195 in AnaPT and with Asn89, Ile169 and Gly173 in LtxC, respectively (Supplementary Fig. 19). These observations would explain why these enzymes can accept not only DMAPP but also longer prenyl pyrophosphates as substrates. In particular, it is noteworthy that both TleC and MpnD have larger active site cavities than these GPP- or FPP-accepting enzymes, which would be the reason why they accept the longer GGPP and GFPP substrates. Most importantly, our results expanded the substrate scope of the enzyme reactions, which will set the stage for further development of new enzymes with novel catalytic functions.

In conclusion, a comparison of the crystal structures of the indolactam-specific TleC and MpnD revealed the intimate structural details of the enzyme-catalysed ‘reverse' prenylation reactions and identified the active site residues governing the selection of the prenyl donors. Furthermore, structure-based enzyme engineering successfully altered the preference for the chain lengths of the prenyl donors, as well as the regio- and stereo-selectivities of the prenylation reactions. These findings provided insights into the catalytic machinery and the strategies towards expanding the catalytic repertoire of the enzymes, to generate designed novel molecular scaffolds for drug discovery.

## Methods

### Materials

(−)-Indolactam V was prepared by using *S. blastmyceticus* according to the published method^14^. DMSPP was purchased from Echelon Bioscience, Inc. Oligonucleotides were obtained from Eurofins Genomics, Inc.

### Compound characterization

High-resolution electrospray ionization mass spectrometry (HRESIMS) spectra were measured with an Agilent 1100 series HPLC-microTOF mass spectrometer (JEOL), using electrospray ionization (ESI). NMR spectra were obtained at 500 MHz (^1^H) and 125 MHz (^13^C) with a JEOL ECX-500 spectrometer.

5-Geranylindolactam V (**4**): ultraviolet: *λ*_max_ 291 nm. HR-MS (ESI): found for [C_27_H_38_N_3_O_2_]^−^ 436.29567: calcd 436.29640. ^1^H NMR (500 MHz, CDCl_3_): *δ* 8.26 (*s*, 1H, NH-1), 7.24 (*d*, 1H, *J*=8.0 Hz, H-7), 7.12 (*d*, 1H, *J*=8.0 Hz, H-6), 7.04 (*d*, 1H, *J*=2.0 Hz, H-2), 5.30 (*t*, 1H, *J*=6.5 Hz, H-2′), 5.15 (*t*, 1H, *J*=7.0 Hz, H-6′), 4.96 (*d*, 1H, *J*=10.5 Hz, NH-10), 4.48 (*m*, 1H, H-9), 3.73 (dd, 1H, *J*=6.5, 15.5 Hz, H-1′), 3.55 (dd, 1H, *J*=6.5, 15.5 Hz, H-1′), 3.43 (dd, 1H, *J*=6.5, 11.0 Hz, H-14), 3.38 (dd, 1H, *J*=6.5, 11.0 Hz, H-14), 3.25 (dd, 1H, *J*=4.5, 14.5 Hz, H-8), 3.05 (*d*, 1H, *J*=10.5 Hz, H-12), 2.81 (dd, 1H, *J*=1.5, 14.5 Hz, H-8), 2.77 (*s*, 3H, H-18), 2.69 (*m*, 1H, H-15), 2.15 (*q*, 2H, *J*=7.0 Hz, H-5′), 2.09 (*t*, 2H, *J*=7.0 Hz, H-4′), 1.75 (*s*, 3H, H-9′), 1.71 (*s*, 3H, H-8′), 1.63 (*s*, 3H, H-10′), 1.31 (*d*, 3H, *J*=7.0 Hz, H-16), 0.99 (*d*, 3H, *J*=6.5 Hz, H-17). ^13^C NMR (125 MHz, CDCl_3_): *δ* 173.4 (C-11), 141.8 (C-4), 138.3 (C-7a), 135.3 (C-3′), 134.2 (C-5), 131.4 (C-7′), 127.9 (C-4a), 124.8 (C-6), 124.7 (C-2 and C-2′), 124.4 (C-6′), 110.4 (C-7), 109.8 (C-3), 73.5 (C-12), 63.1 (C-14), 55.4 (C-9), 39.8 (C-4'), 36.2 (C-18), 30.8 (C-1'), 29.6 (C-8), 26.6 (C-5'), 26.4 (C-15), 25.7 (C-8′), 20.9 (C-17), 19.8 (C-16), 17.7 (C-10′), 16.2 (C-9′). For full NMR spectra of compound **4**, see Supplementary Fig. 20.

5-Farnesylindolactam V (**5**): ultraviolet: *λ*_max_ 291 nm. HR-MS (ESI): found for [C_32_H_46_N_3_O_2_]^−^ 504.35752: calcd 504.35900. ^1^H NMR (500 MHz, CDCl_3_): *δ* 8.17 (*s*, 1H, NH-1), 7.21 (*d*, 1H, *J*=8.5 Hz, H-7), 7.10 (*d*, 1H, *J*=8.5, H-6), 7.02 (*s*, 1H, H-2), 5.29 (*t*, 1H, *J*=7.0 Hz, H-2′), 5.14 (*t*, 1H, *J*=6.5 Hz, H-6′), 5.09 (*m*, 1H, H-10′), 4.93 (*d*, 1H, *J*=10.5 Hz, NH-10), 4.46 (*m*, 1H, H-9), 3.71 (dd, 1H, *J*=7.0, 15.5 Hz, H-1′), 3.53 (dd, 1H, *J*=7.0, 15.5 Hz, H-1′), 3.41 (dd, 1H, *J*=6.5, 12.0 Hz, H-14), 3.36 (dd, 1H, *J*=6.5, 12.0 Hz, H-14), 3.23 (dd, 1H, *J*=4.5, 14.5 Hz, H-8), 3.02 (d, 1H, *J*=11.0 Hz, H-12), 2.79 (d, 1H, *J*=14.5, H-8), 2.74 (*s*, 3H, H-18), 2.66 (*m*, 1H, H-15), 2.14 (*m*, 2H, H-5′), 2.07 (*m*, 2H, H-4′), 2.06 (*m*, 2H, H-9′), 1.97 (*m*, 2H, H-8′), 1.73 (*s*, 3H, H-13′), 1.67 (*s*, 3H, H-12′), 1.60 (*s*, 3H, H-14′), 1.59 (*s*, 3H, H-15′), 1.29 (*d*, 3H, *J*=7.0 Hz, H-16), 0.97 (*d*, 3H, *J*=6.5 Hz, H-17). ^13^C NMR (125 MHz, CDCl_3_): *δ* 173.4 (C-11), 141.9 (C-4), 138.3 (C-7a), 135.5 (C-3′), 135.1 (C-7′), 134.3 (C-5), 131.3 (C-11′), 127.9 (C-4a), 124.8 (C-6), 124.7 (C-2 and C-2′), 124.4 (C-10′), 124.2 (C-6′), 110.3 (C-7), 109.9 (C-3), 73.5 (C-12), 63.1 (C-14), 55.4 (C-9), 39.8 (C-4′ and C-8′), 36.2 (C-18), 30.8 (C-1′), 29.6 (C-8), 26.8 (C-9′), 26.6 (C-5′), 26.4 (C-15), 25.7 (C-12′), 20.9 (C-17), 19.8 (C-16), 17.7 (C-15′), 16.3 (C-13′), 16.1 (C-14′). For full NMR spectra of compound **5**, see Supplementary Fig. 21.

### Enzyme expression and purification of the TleC and MpnD enzymes

The DNA encoding full-length TleC was amplified from the genome of *S. blastmyceticus* by PCR, using 5′-CATATGGAGTCCGCAGGTCCCGGAACC-3′ as the sense primer, which introduces an NdeI restriction site, and 5′-AAGCTTCGCCTCCCGGTAGACCCCCGGC-3′ as the antisense primer, which introduces a HindIII restriction site. The DNA encoding full-length MpnD from *M. thermotolerans* was purchased from Eurofins Genomics, Inc., and has NdeI and XhoI restriction sites at the 5′- and 3′-termini, respectively. The DNA fragments were digested with NdeI/HindIII (TleC) and NdeI/XhoI (MpnD), and ligated into the NdeI/HindIII (TleC) and NdeI/XhoI (MpnD) sites of the digested pET22b vector (Merck Millipore), for expression as fusion proteins with a His_6_-tag at the carboxy terminus. After confirmation of the sequence, the pET22b vectors encoding full-length TleC and MpnD were transformed into *E. coli* BLR (DE3). The cells harbouring the plasmids were cultured to an OD_600_ of 0.6 in Luria-Bertani medium containing 100 μg ml^−1^ ampicillin at 37 °C. Isopropyl β-D-1-thiogalactopyranoside was then added to a final concentration of 0.2 mM, to induce gene expression, and the cultures were incubated further for 16 h at 20 °C.

All of the following procedures were performed at 4 °C. The *E. coli* cells were harvested by centrifugation at 5,000 *g* and resuspended in 50 mM Tris–HCl buffer pH 7.5, containing 200 mM NaCl, 5% (v/v) glycerol and 5 mM imidazole (buffer A). The cells were disrupted by sonication and the lysate was centrifuged at 12,000 *g* for 30 min. The supernatant was loaded onto a COSMOGEL His-Accept (Nacalai Tesque) column, equilibrated with buffer A. After washing the resin with buffer A containing 10 mM imidazole, the recombinant TleC protein was subsequently eluted with buffer A containing 300 mM imidazole. The protein solution was concentrated to 5 ml, purified to homogeneity by gel-filtration chromatography on a HiLoad 16/60 Superdex 200 pg column (GE Healthcare), and concentrated to 10 mg ml^−1^ in 20 mM Tris–HCl buffer pH 7.5, containing 150 mM NaCl and 1 mM dithiothreitol. The protein concentration was calculated by measuring ultraviolet absorption at A_280_ (ref. 33). MpnD was purified by the same method as for TleC, except Tris–HCl buffer pH 8.5 instead of Tris–HCl buffer pH 7.5.

The selenomethionine-labelled TleC protein was overexpressed in *E. coli* strain B834 (DE3). The cells were grown at 37 °C in 8 l of LeMaster medium, containing 25 mg l^−1^ L-selenomethionine, Sigma Kao and Michayluk Vitamin Solution (Sigma–Aldrich) and 100 μg ml^−1^ ampicillin, until the cell density reached an OD_600_ of 1.2. At this point, 0.2 mM isopropyl β-D-1-thiogalactopyranoside was added to induce the expression of the selenomethionine-labelled and C-terminally His_6_-fused TleC, and the growth was continued for 18 h at 20 °C. The TleC (SeMet) protein was purified as described above.

### Enzyme assays

TleC or MpnD (10 μg) was incubated with 100 μM of indolactam V and 200 μM of prenyl pyrophosphates (DMAPP, GPP or FPP), in a final volume of 100 μl of 50 mM Tris–HCl buffer containing 150 mM NaCl pH 7.0, at 42 °C for 10 min. When FPP (TleC reaction), GGPP and GFPP were used as substrates, the enzyme reactions were performed at 37 °C for 18 h. The reactions were stopped by the addition of 100 μl of MeOH. After the aggregated protein was removed by centrifugation, an aliquot of the sample (100 μl) was subjected to HPLC on a Mightysil RP-18 GP column (4.6 × 250 mm) (KANTO KAGAKU), at a flow rate of 1.0 ml min^−1^. Gradient elution was performed with H_2_O and CH_3_CN, both containing 0.1% trifluoroacetic acid: 0–6 min, 50–65% CH_3_CN; 6–10 min, 65–100% CH_3_CN; and 10–25 min, 100% CH_3_CN. The absorbance of the enzyme reaction products was monitored at 303 nm. For the LC–MS analysis, the samples were prepared in the same manner and analysed by LC–MS on a COSMOSIL C18-MS-II column (2 × 75 mm) (Nacalai Tesque), at a flow rate of 0.2 ml min^−1^. Gradient elution was performed with H_2_O and CH_3_CN, both containing 0.1% acetic acid: 0–3 min, 30% CH_3_CN; 3–15 min, 30–100% CH_3_CN; and 15–20 min, 100% CH_3_CN. The enzyme reaction products was purified by preparative HPLC on a COSMOSIL 5C18-MS-II column (10 × 250 mm) (Nacalai Tesque), at a flow rate of 3.0 ml min^−1^. Isocratic elution was performed with 60% CH_3_CN/H_2_O containing 0.1% acetic acid.

To determine the kinetic parameters of wild-type TleC and MpnD with **1**, the assays contained prenyl pyrophosphate (TleC: GPP, MpnD: DMAPP, 200 μM), **1** (0.1–20 μM) and purified TleC and MpnD. The reactions for the determination of the kinetic parameters were incubated for 3 min at 42 °C. The kinetic parameters of wild-type TleC and MpnD, and their mutants with DMAPP, GPP and FPP, were determined with **1** (150 μM), DMAPP (1–150 μM) and either GPP (1–150 μM) or FPP (5–300 μM). The reaction mixtures were incubated for 5 min at 42 °C. The enzymatic reactions were stopped by the addition of MeOH (100 μl) and analysed by HPLC. The authentic concentrations of the products were calculated by the total peak areas of the products. The experiments were performed in triplicate. The *K*_M_ and *k*_cat_ values were calculated using GraphPad Prism 6 for Mac OS X (GraphPad Software, Inc., La Jolla, CA).

### Crystallization and structure determination

Well-diffracting selenomethionine-labelled TleC and wild-type TleC crystals were obtained at 20 °C, in 100 mM MES-NaOH (pH 6.5) containing 1,700–1,785 mM (NH_4_)_2_SO_4_, with 10 mg ml^−1^ of the SeMet-labelled purified TleC solution, by using the sitting-drop vapour-diffusion method. The crystals of wild-type of MpnD were obtained at 20 °C, in 100 mM Tris–HCl pH 8.5 containing 1,610 mM (NH_4_)_2_SO_4_, with 15 mg ml^−1^ of purified MpnD solution, by using the sitting-drop vapour-diffusion method. The crystals were transferred into the reservoir solution with 18-22% (v/v) glycerol as a cryoprotectant and then flash cooled at −173 °C in a nitrogen-gas stream. X-ray diffraction data sets were collected at BL-17A, BL-5A and NW12A at the Photon Factory, Tsukuba, Japan. The ternary complex containing both substrates was prepared by incubating the TleC and MpnD crystals for 1 min at 20 °C with 5 mM **1** and 5 mM DMSPP. We used wavelengths of 0.9788 Å for the SeMet-labelled TleC, based on the fluorescence spectrum of the Se K absorption edge^34^, 0.9800 Å for TleC and 1.0000 Å for MpnD, for data collection.

The diffraction data for TleC SeMet were processed and scaled using the *XDS* programme package^35^. The Se sites were determined with *AutoSol* in *PHENIX*^36^,^37^. The sites were refined and the initial phases were calculated with *AutoBuild* in *PHENIX*^36^,^38^. The structure was modified manually with *Coot*^39^ and refined with *PHENIX*^36^,^40^. The initial phases of the TleC ternary complex structure and the MpnD ternary complex structure were determined by molecular replacement, using the structure of TleC SeMet as the search model. Molecular replacement was performed with *Phaser* in *PHENIX*^36^,^41^. The refinements were performed in the same manner as for TleC SeMet. The final crystal data and intensity statistics are summarized in Table 2. A structural similarity search was performed by using the Dali programme^42^. The cavity volumes were calculated with the programme CASTP (http://cast.engr.uic.edu/cast/). All crystallographic figures were prepared with PyMOL (DeLano Scientific, http://www.pymol.org).

### Site-directed mutagenesis

The plasmids expressing the mutants of TleC (A173M, W97Y/A173M and W97Y/F170W/A173M) and MpnD (M159A, Y80W/M159A and Y80W/W157F/M159A) were constructed with a QuikChange Site-Directed Mutagenesis Kit (Stratagene), according to the manufacturer's protocol. The mutant proteins were expressed and purified in the same manner as for the wild-type enzymes and the enzyme reactions were performed with the same methods as for the wild-type enzymes.

### Molecular modelling

The three-dimensional model of GPP was generated by the Chem3D Ultra 13 programme (CambridgeSoft). Docking calculations were performed using AutoDock4 (ref. 43). The mutant models were constructed by manually swapping of the GPP in the active site of the TleC mutant model structure by using *Coot* and the energy minimization calculation by simulated annealing with *PHENIX* was then performed. The parameters of the intermediates for the energy minimization calculation were obtained from the PRODRG server.

## Additional information

**Accession codes:** Protein Data Bank (PDB): The coordinates and the structure factor amplitudes for the selenomethionine-labelled TleC, the TleC apo structure, the TleC complexed with (−)-indolactam V and DMSPP, the MpnD apo structure and the MpnD complexed with (−)-indolactam V and DMSPP were deposited under accession codes 4YZJ, 4YZK, 4YZL, 4YL7 and 4YLA, respectively.

**How to cite this article:** Mori, T. *et al.* Manipulation of prenylation reactions by structure-based engineering of bacterial indolactam prenyltransferases. *Nat. Commun.* 7:10849 doi: 10.1038/ncomms10849 (2016).

## Supplementary Material

Supplementary Information Supplementary Figures 1-21

## Figures and Tables

**Figure 1:**
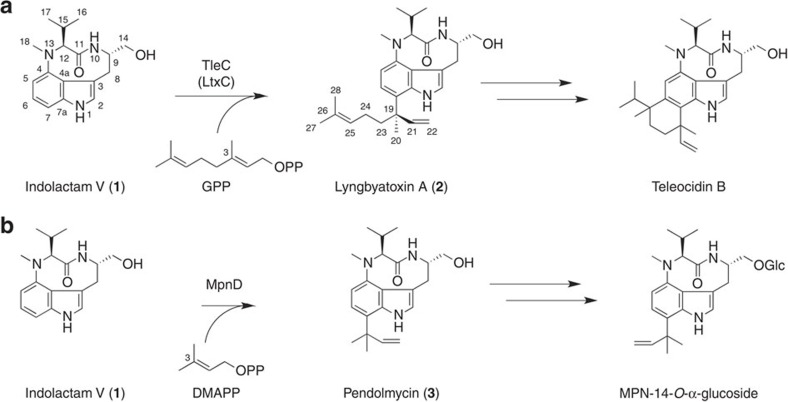
Reaction schemes of indole prenyltransferases. Reaction schemes for the conversion of (−)-indolactam V (**1**) into (**a**) lyngbyatoxin A (**2**) by *S. blastmyceticus* TleC and *M. producens* LtxC, and (**b**) pendolmycin (**3**) by *M. thermotolerans* MpnD.

**Table 1:** The steady-state enzyme kinetics values of the wild-type and mutants of (A) *S. blastmyceticus* TleC and (B) *M. thermotolerans* MpnD.

**(A) TleC**	***k***_**cat**_ **(min)**	***K***_**M**_ **(μM)**	***k***_**cat**_**/*****K***_**M**_ **(s**^**−1**^ **mM**^**−1**^**)**	**Product**
*TleC WT*				
DMAPP	1.5±0.1	21.7±4.3	1.1	**3**
GPP	15.0±1.5	9.5±3.3	26.4	**2**:**4**=26:1
				
*TleC W97Y*				
DMAPP	24.1±0.8	11.3±1.6	35.5	**3**
GPP	10.1±0.4	7.7±1.0	21.8	**2**:**4**=24:1
				
*TleC A173M*				
DMAPP	3.9±0.1	24.6±3.0	2.6	**3**
GPP	n.d.	n.d.	ND	**2**:**4**:**8**=1:0.4:0.6
				
*TleC W97Y/A173M*				
DMAPP	2.0±0.1	41.2±5.6	0.8	**3**
GPP	0.7±0.1	34.4±9.3	0.3	**2**:**4**:**8**=1:0.6:2.3
				
*TleC W97Y/F170W/A173M*				
DMAPP	2.6±0.2	46.6±8.2	0.9	**3**
GPP	0.7±0.0	46.8±6.7	0.3	**2**:**4**:**8**=1:1:2.9
				
*(B)*				
* MpnD WT*				
* *DMAPP	52.9±2.6	4.8±1.0	183.0	**3**
* *GPP	2.6±0.2	26.0±4.9	1.7	**2**:**4**=1:3
* *FPP	5.2±0.5	95.5±17.1	0.9	**5**
* MpnD Y80W*				
* *DMAPP	7.1±0.3	15.3±2.5	7.7	**3**
* *GPP	5.4±0.1	9.7±1.1	9.3	**2**:**4**=1:2.3
* *FPP	4.8±0.7	148.3±46.8	0.5	**5**
* MpnD M159A*				
* *DMAPP	30.3±2.0	24.2±4.2	20.9	**3**
* *GPP	4.3±0.2	9.1±1.2	7.9	**2**:**4**=15.6:1
* *FPP	10.0±0.9	48.7±14.6	3.4	**5**
* MpnD Y80W/M159A*				
* *DMAPP	4.5±0.2	4.3±0.7	17.0	**3**
* *GPP	5.8±0.7	33.0±11.0	2.9	**2**:**4**=1:1.9
* *FPP	5.6±0.4	96.5±14.3	1.0	**5**

DMAPP, dimethylallyl pyrophosphate; FPP, farnesyl pyrophosphate; GPP, geranyl pyrophosphate.

**Table 2:** Data collection, phasing and refinement statistics.

	**TleC SeMet**	**TleC apo**	**TleC complex**	**MpnD apo**	**MpnD complex**
*Data collection*					
Space group	P2_1_2_1_2_1_	P2_1_2_1_2_1_	P2_1_2_1_2_1_	P2_1_2_1_2_1_	P2_1_2_1_2_1_
Cell dimensions					
*a*, *b*, *c* (Å)	41.9, 73.9, 110.4	42.0, 74.1, 111.0	42,4, 74.1, 112.2	46.7, 53.7, 132.7	46.8, 54.1, 132.2
Resolution (Å)	50.0–2.1 (2.23–2.11)*	50.0–1.95 (2.07–1.95)*	50.0–2.1 (2.22–2.10)*	50.0–1.6 (1.70–1.60)*	50.0–1.4 (1.48–1.40)*
*R*_merge_ (%)	10.7 (70.4)	11.4 (73.5)	8.4 (51.9)	4.9 (31.5)	4.9 (45.2)
*I*/σ*I*	15.4 (3.3)	13.4 (3.1)	22.7 (4.4)	25.0 (5.8)	22.8 (4.0)
Completeness (%)	99.4 (96.5)	99.5 (97.1)	99.8 (98.6)	99.6 (98.3)	99.7 (99.2)
Redundancy	7.7 (7.4)	7.2 (7.2)	7.2 (7.1)	7.2 (7.0)	6.4 (6.3)
					
*Refinement*					
Resolution (Å)	44.2–2.1	44.4–2.0	44.7–2.1	34.1–1.6	41.9–1.4
No. reflections	20415	25878	21401	44640	66871
*R*_work_/*R*_free_ (%)	18.8/23.5	18.6/23.2	18.9/23.7	18.0/20.4	17.8/20.3
No. atoms					
Protein	2606	2652	2766	2791	2816
Ligand/ion	—	—	36	—	51
Water	125	224	142	363	429
*B*-factors					
Protein	30.5	26.6	28.6	18.5	15.6
Ligand/ion	—	—	25.1	—	20.8
Water	34.3	32.4	30.7	27.3	25.6
Root mean square deviations					
Bond lengths (Å)	0.009	0.007	0.009	0.006	0.009
Bond angles (°)	1.211	1.032	1.182	1.127	1.247

^*^One crystal was used for data collection. Values in parentheses are for highest-resolution shell.
