# Supplementary material for: Manipulation of prenylation reactions by structure-based engineering of bacterial indolactam prenyltransferases
Source: Nat Commun. 2016 Mar 8;7:10849. doi: 10.1038/ncomms10849 (PMC4786772; doi:10.1038/ncomms10849)
Supplement: Supplementary Information — Supplementary Figures 1-21 [file ncomms10849-s1.pdf]

Supplementary Figures

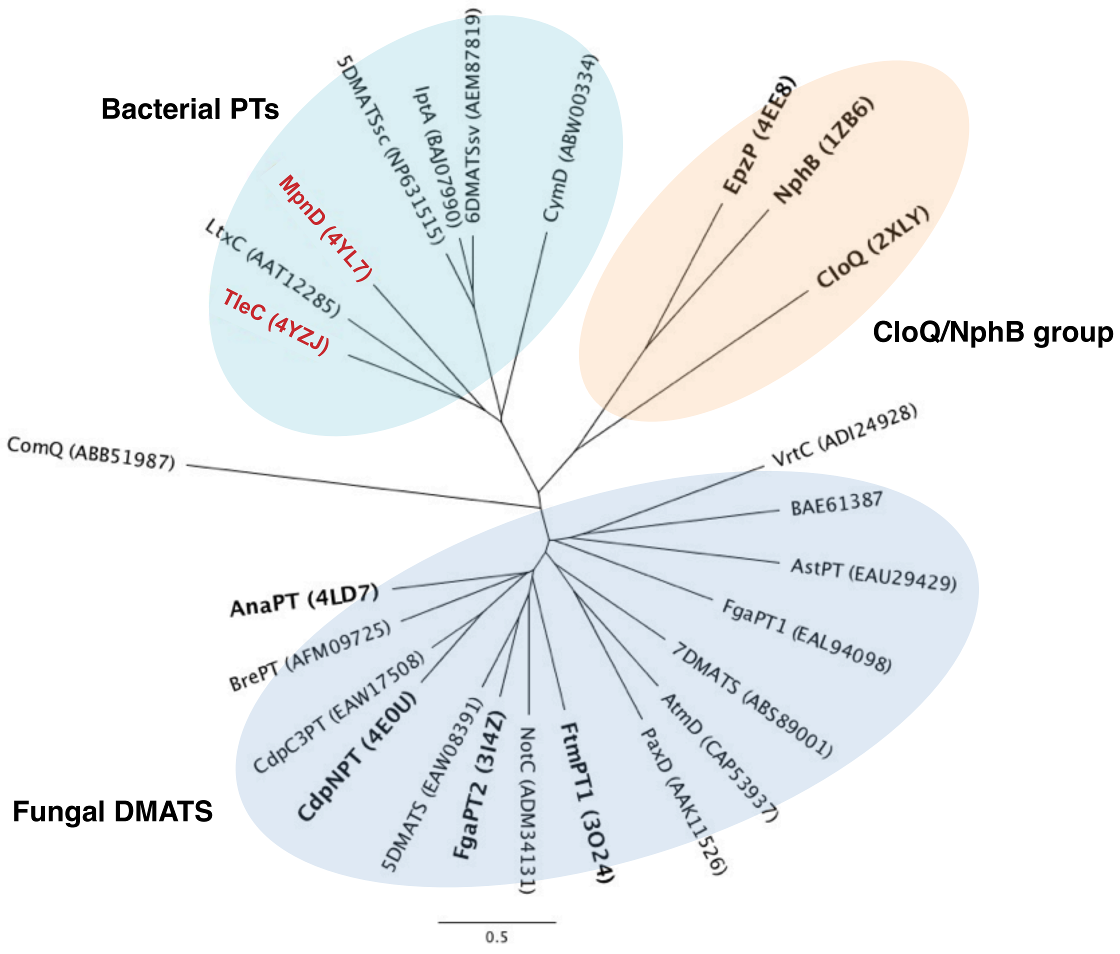

Supplementary Figure 1. Phylogenetic tree analysis of soluble indole and aromatic prenyltransferases. The phylogenetic tree was created by using the programs ClustalX2 (http://www.clustal.org/). A peptide indole prenyltransferase ComQ is included as an outgroup. Structurally characterized PTs are in bold style. The accession numbers or pdb codes of the enzymes are given in parenthesis.

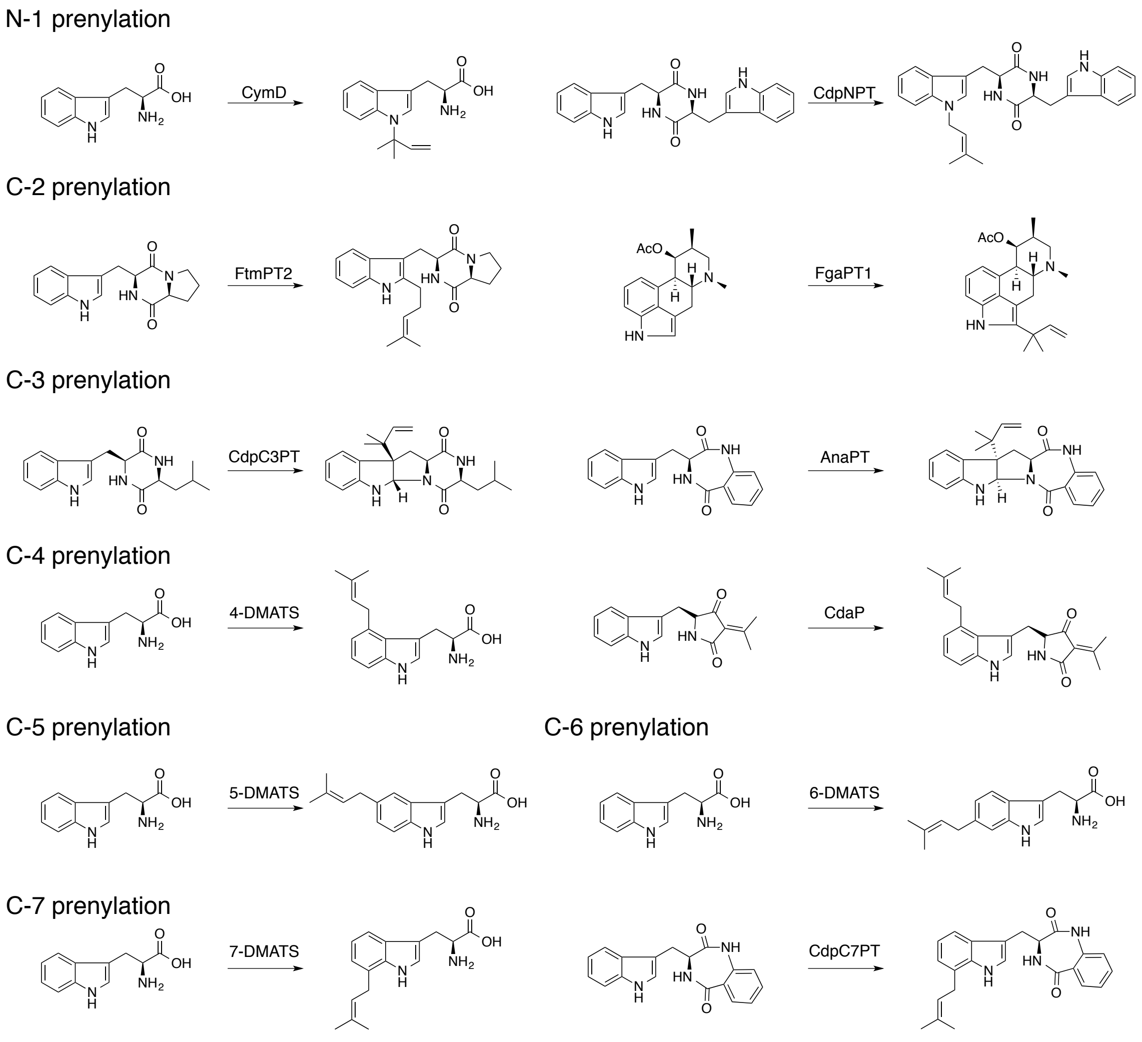

Supplementary Figure 2. The reactions of indole prenyltransferases with different prenylation positions.

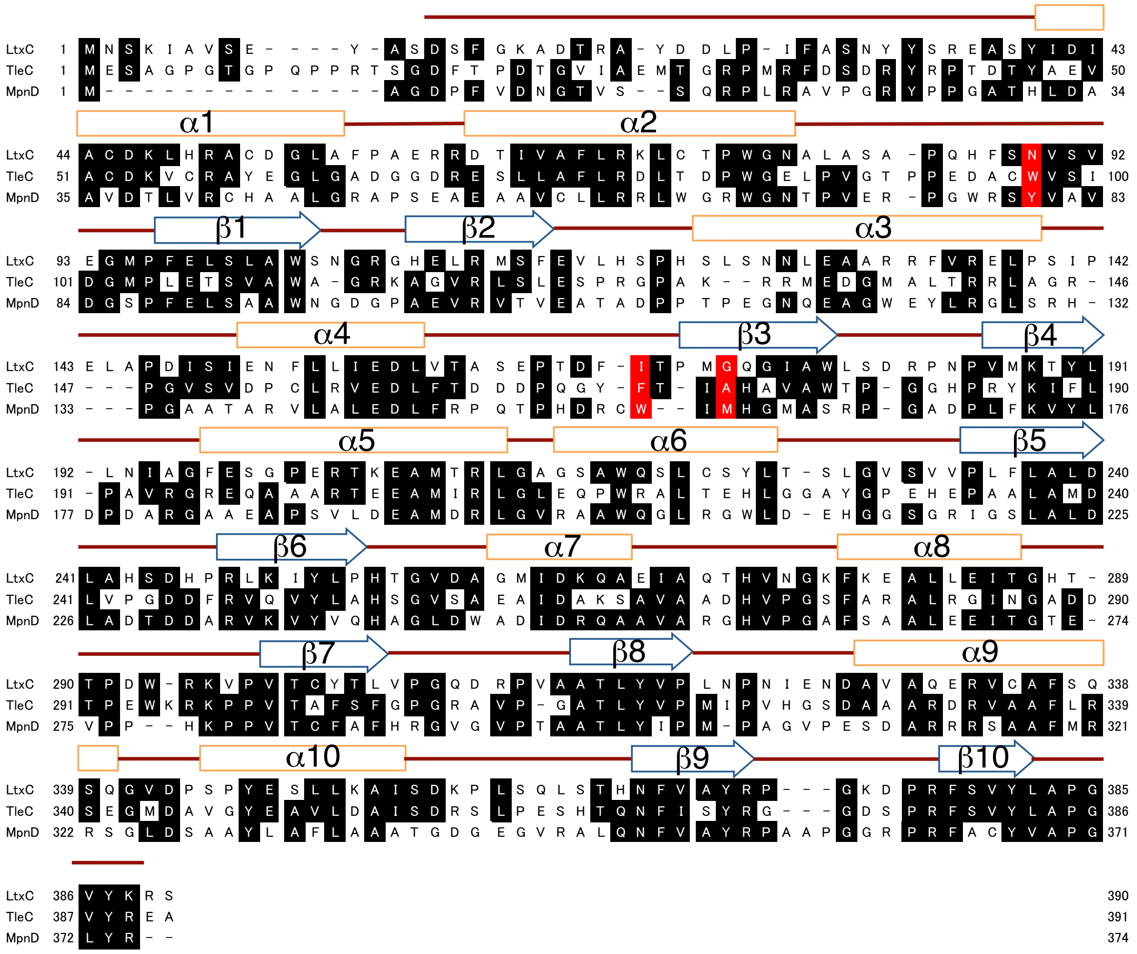

Supplementary Figure 3. Comparison of the primary sequences of TleC, MpnD, and LtxC. The secondary structures of TleC and MpnD are delineated as follows: α-helices (orange rectangles), β-strands (blue arrows), and loops (red, bold lines). The three important residues discussed in this report are colored red. TleC is from *Streptomyces blastmyceticus* (BAP27943), MpnD is from *Marinactinospora thermotolerans* (AFO85455) and LtxC is from *Moorea producens* (AAT12285).

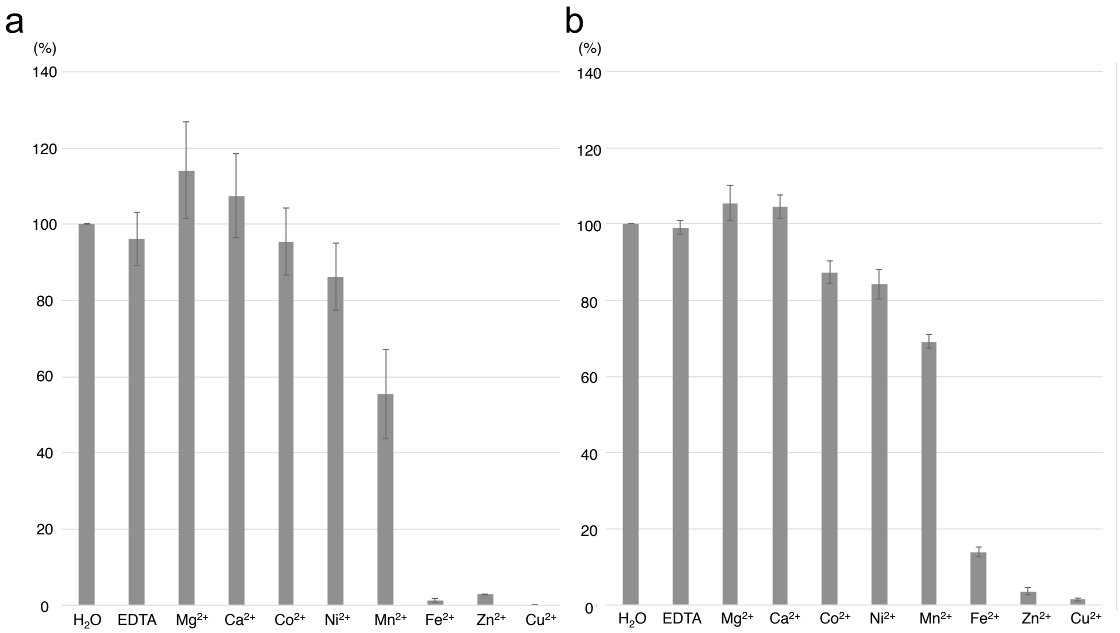

Supplementary Figure 4. The result of metal-ion dependency. The effects of metal ions on the prenylation activities of (a) TleC and (**b**) MpnD (n = 3).

Prenyl acceptors

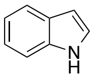
indole

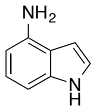
4-aminoindole

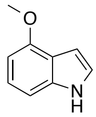
4-methoxyindole

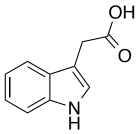
indole acetic acid

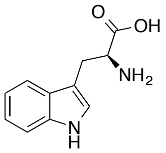
L-tryptophan

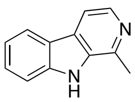
harman

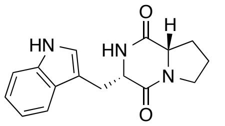
brevianamide F

Prenyl donors

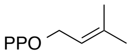
DMAPP

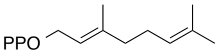
GPP

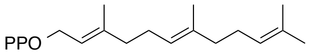
FPP

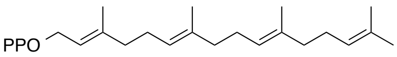
GGPP

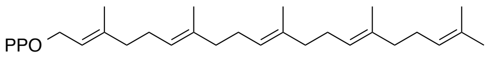
GFPP

Supplementary Figure 5. List of substrate analogs. The substrate analogs used in this study.

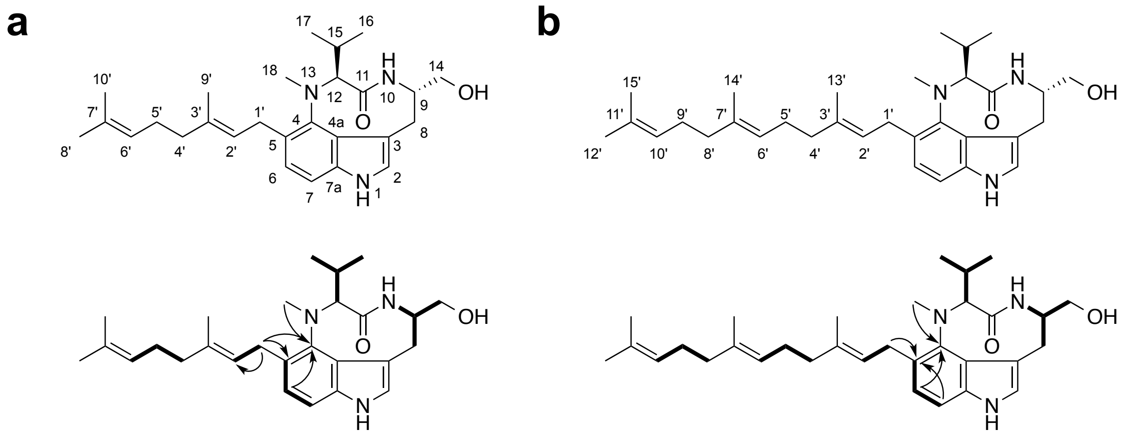

Supplementary Figure 6. The key HMBC and key COSY correlations in 4 and 5. The numbering, HMBC, and COSY correlations in (**a**) **4** and (**b**) **5**. Arrows indicate the key HMBC correlations and bold bonds represent the key COSY correlations.

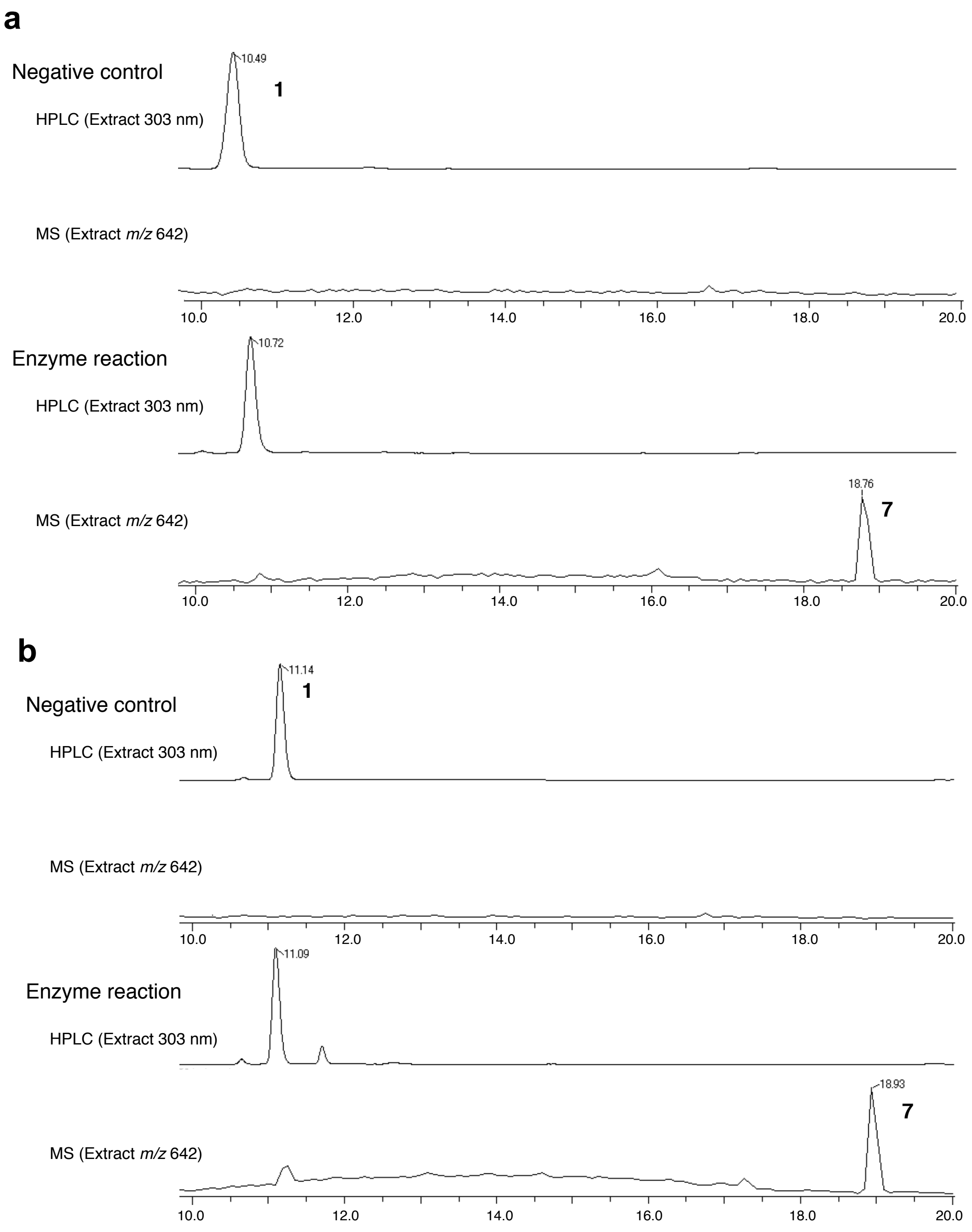

Supplementary Figure 7. The enzymatic reactions of TleC and MpnD with GFPP as a substrate. HPLC elution profiles of the enzyme reaction products of (**a**) TleC and (**b**) MpnD from **1** and GFPP.

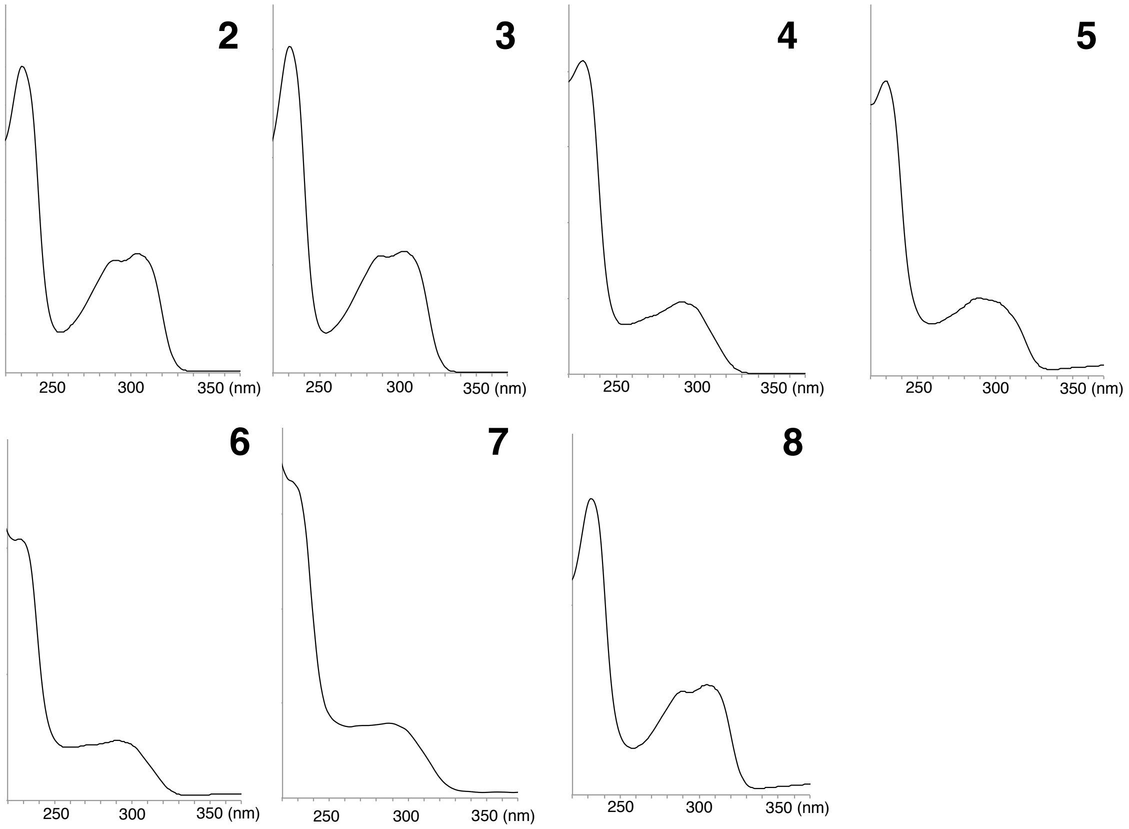

Supplementary Figure 8. The UV spectra of the compounds.

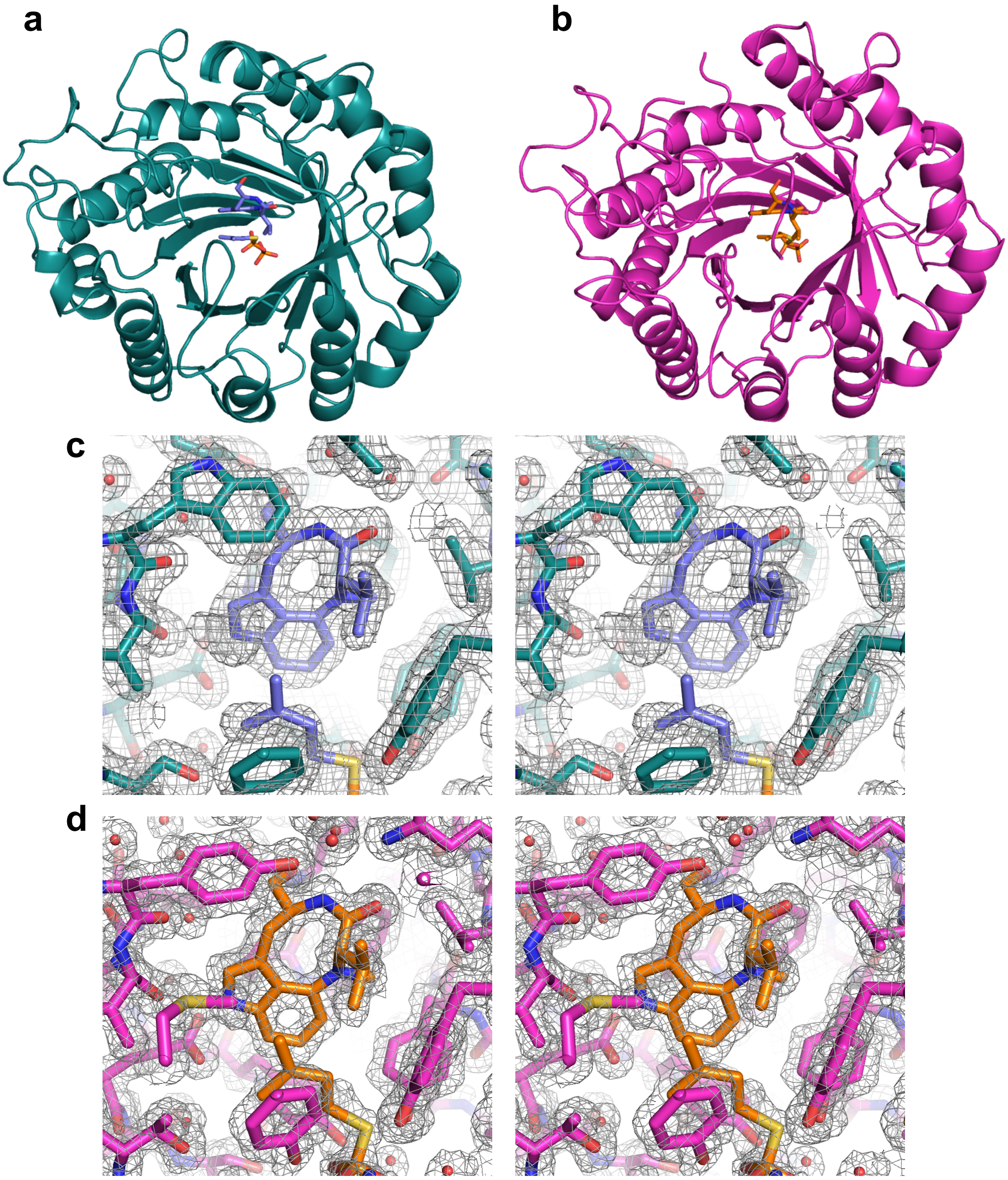

Supplementary Figure 9. The overall structures of TleC, MpnD. Comparison of the overall structures of (**a**) TleC and (**b**) MpnD. (**c**) Stereo view of the final complex structure of TleC, **1**, and DMSPP, fitted in the final $2F_O$-F_C electron density map contoured at 1 σ. (**d**) Stereo view of the final complex structure of MpnD, **1**, and DMSPP, fitted in the final $2F_O$-F_C electron density map contoured at 1 σ. The substrates (**1** and DMSPP) in TleC and MpnD are depicted by blue and orange stick models, respectively.

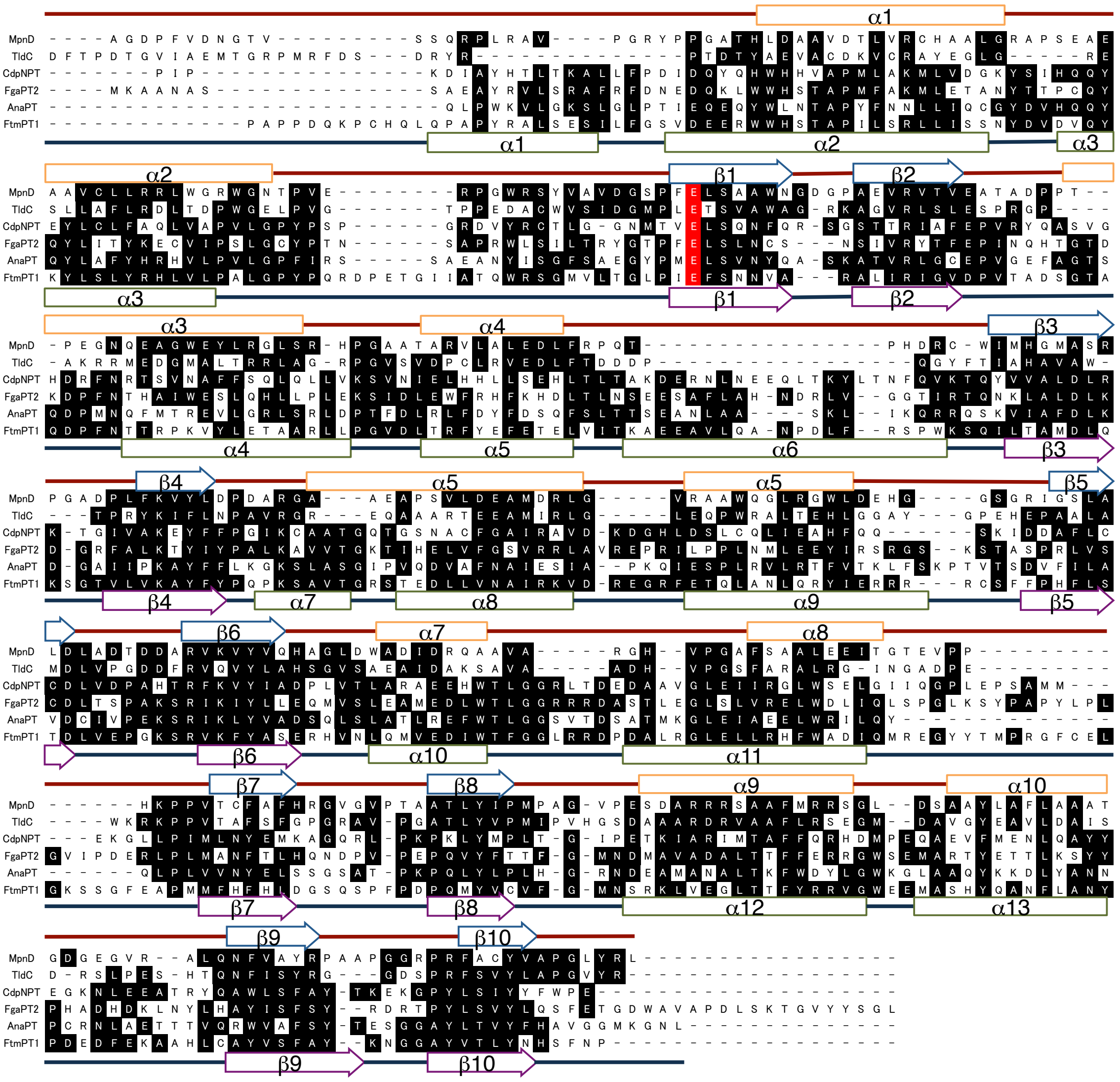

Supplementary Figure 10. The structure-based sequence alignment of TleC, MpnD, and the other indole PTs. The secondary structures of TleC and MpnD are delineated as follows: α-helices (orange rectangles), β-strands (blue arrows), and loops (red, bold lines). The secondary structures of known indole PTs are delineated as follows: α-helices (green rectangles), β-strands (purple arrows), and loops (navy blue, bold lines). The catalytic residue glutamic acid is colored red.

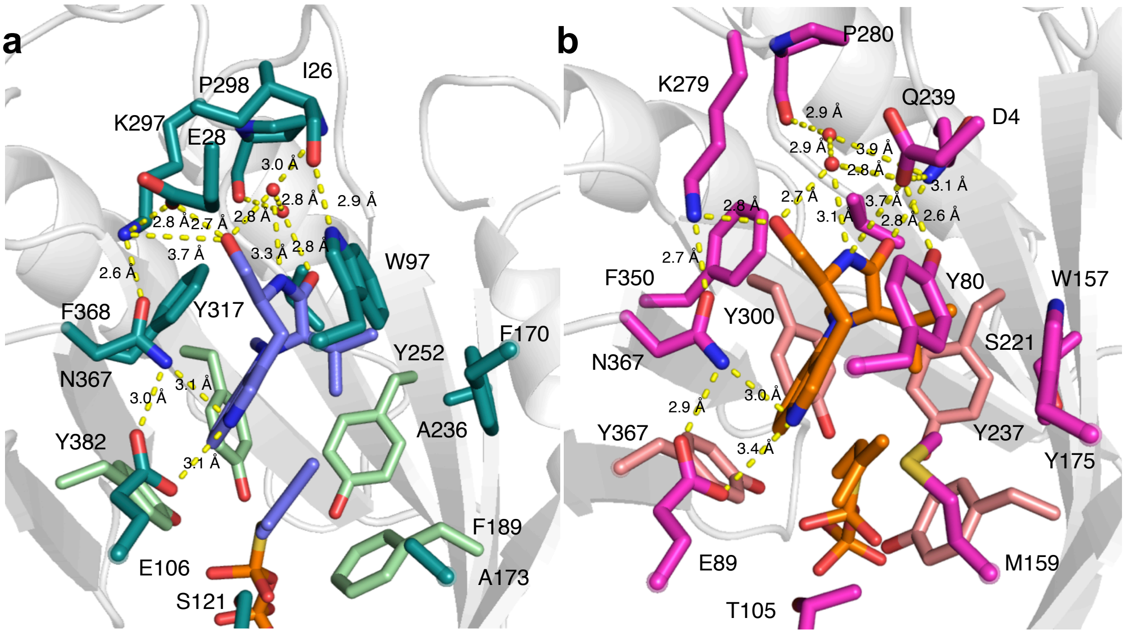

Supplementary Figure 11. Comparison of the binding modes of 1 in TleC and MpnD. Close-up views of the **1** binding site in (**a**) the TleC-**1**-DMSPP ternary complex and (**b**) the MpnD-**1**-DMSPP ternary complex. The substrates (**1** and DMSPP) in TleC and MpnD are depicted by a blue and orange stick models, respectively. The amino acid residues located in the diphosphate binding site and the binding site of **1** are represented by limegreen and green stick models in TleC and salmon and magenta stick models in MpnD, respectively. Dashed yellow lines represent hydrogen bonds.

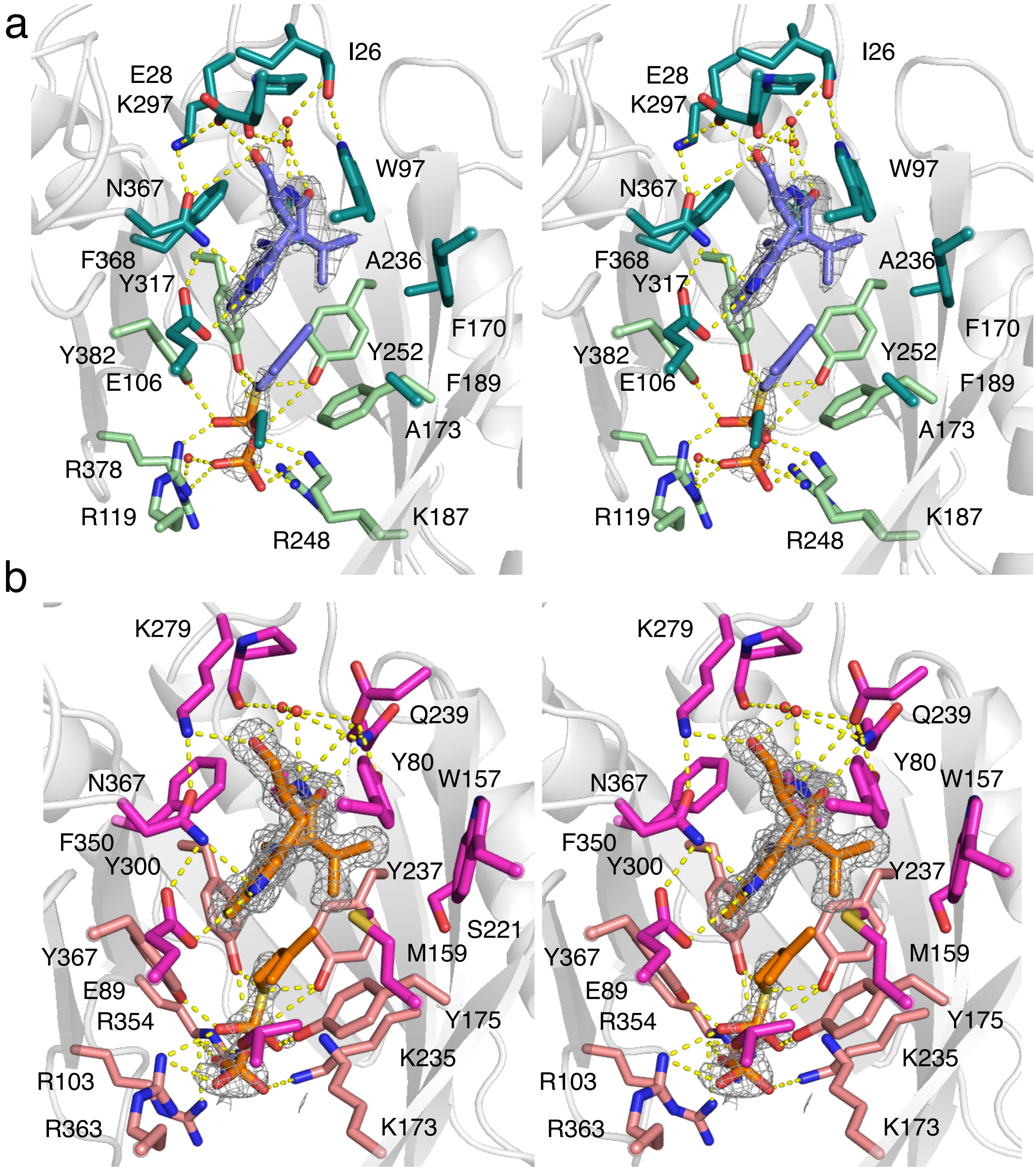

Supplementary Figure 12. Stereo view of Figure 3. (a) A ternary complex structure of TleC with indolactam V and DMSPP and (b) a ternary complex structure of MpnD with indolactam V and DMSPP.

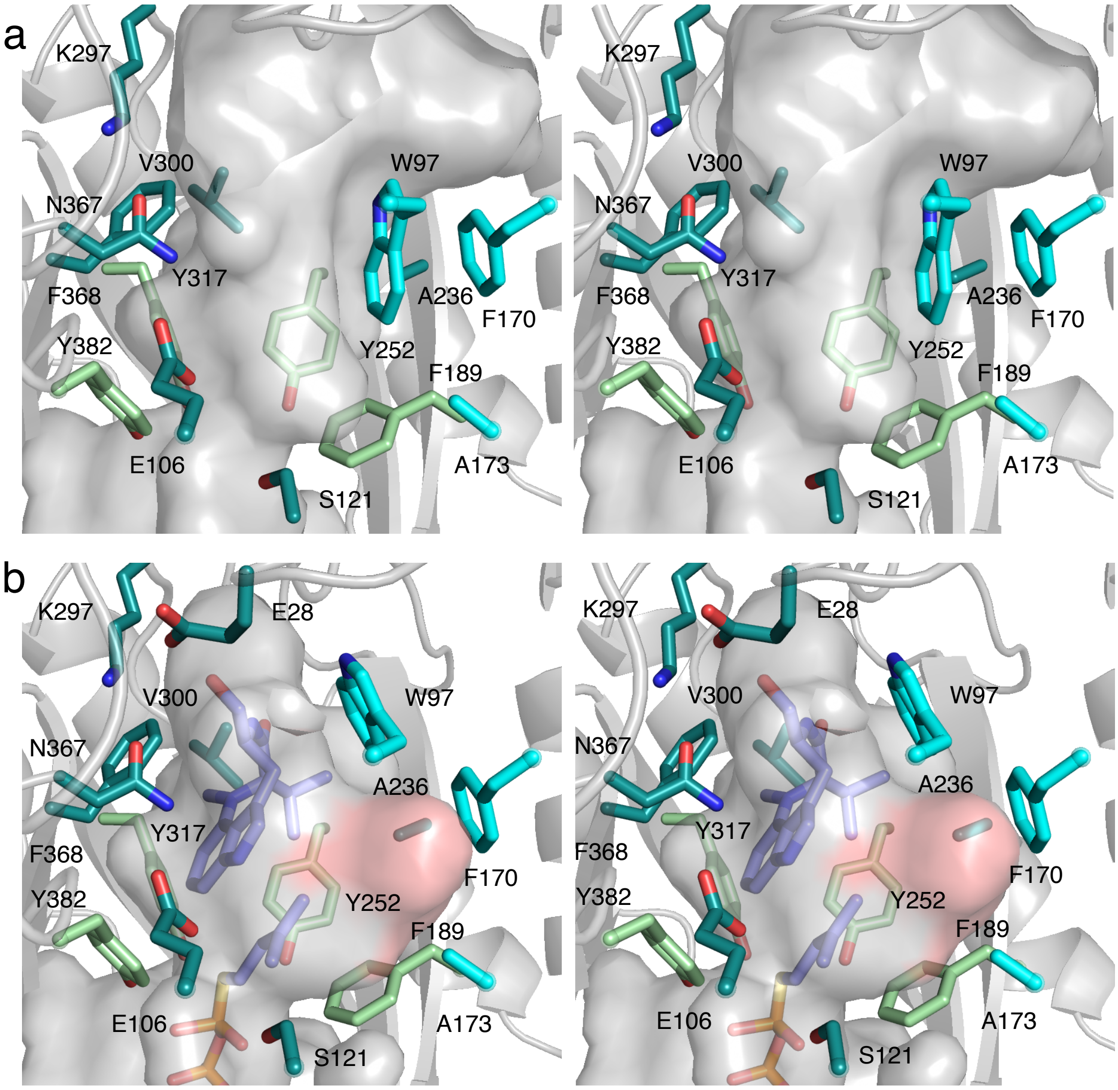

Supplementary Figure 13. Stereo view of Figure 4. (a) A apo structure of TleC and (b) a ternary complex structure of TleC with indolactam V and DMSPP.

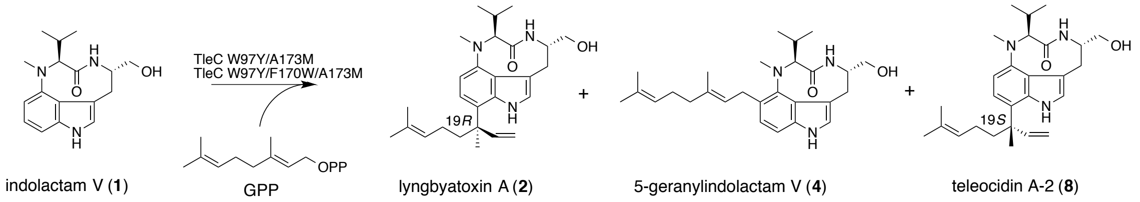

Ratio = 1 : 1 : 2.9

Supplementary Figure 14. The enzyme reactions of the TleC W97/A173M and W97Y/F170W/A173M mutants.

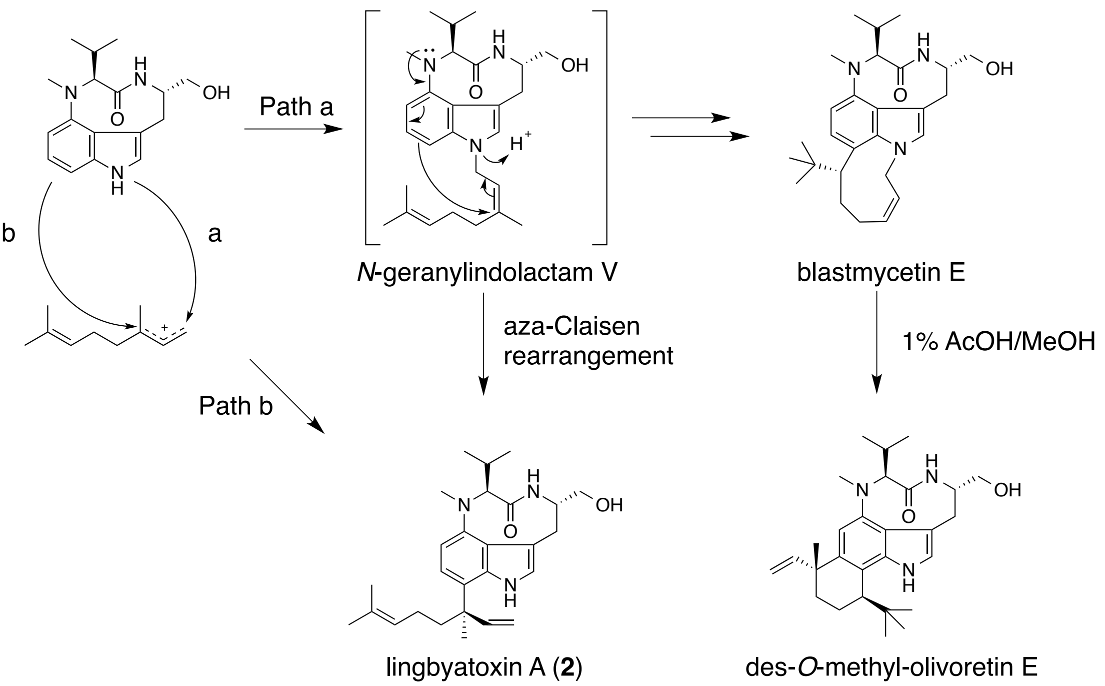

Supplementary Figure 15. Proposed biosynthetic pathways of 2. (Path a) The biosynthetic pathway of **2** through *N*-geranylindolactam V. **(**Path b) The direct prenylation of GPP to C-7 of **1** to produce **2**.

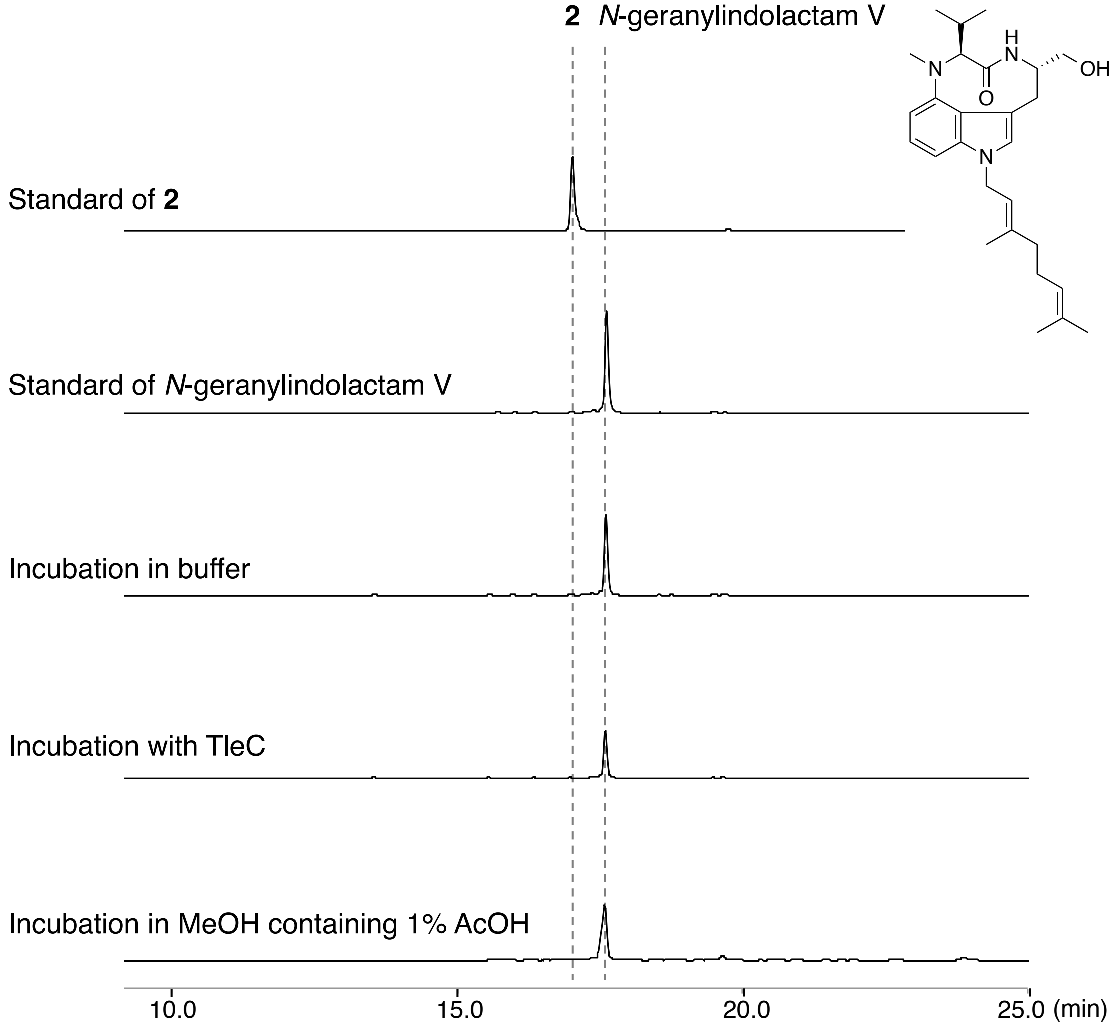

Supplementary Figure 16. The reaction of *N*-geranylindolactam V with TleC. HPLC elution profiles of the standard of **2**, the standard of *N*-geranylindolactam V, *N*-geranylindolactam V after an incubation in buffer containg Tris-HCl (pH 7.0), *N*-geranylindolactam V after incubation with TleC, and *N*-geranylindolactam V after an incubation in MeOH containing 1% AcOH.

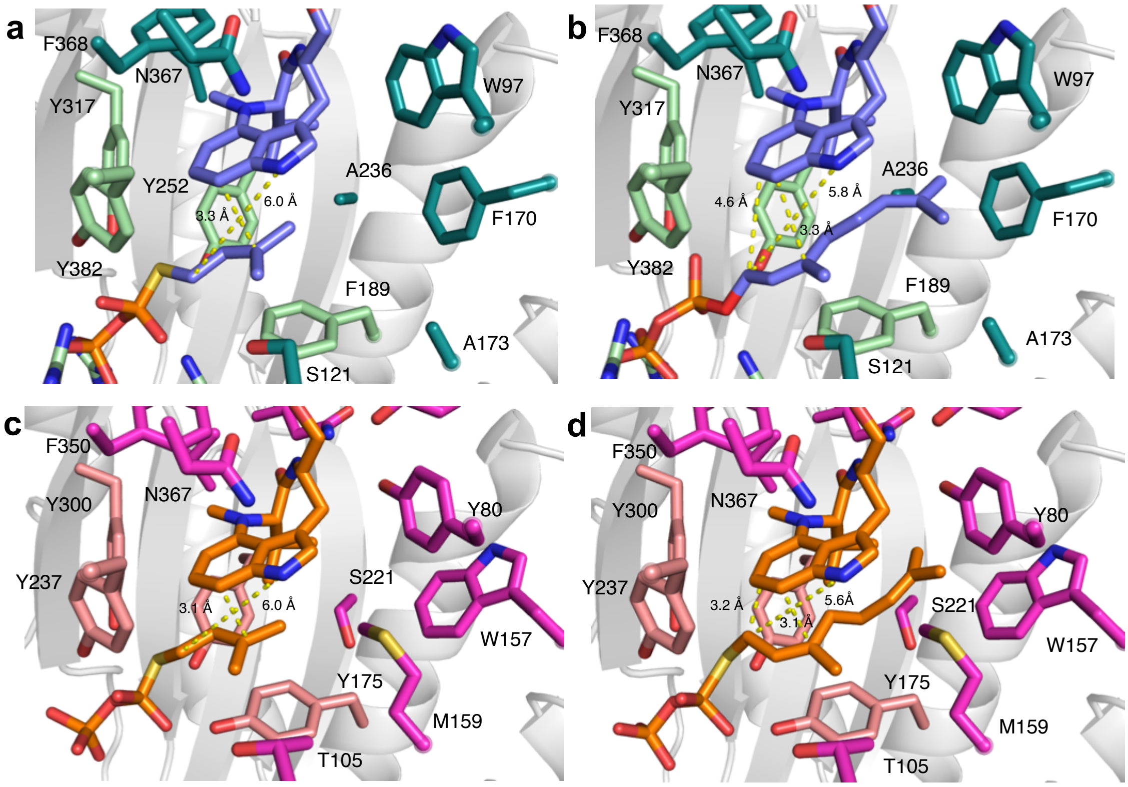

Supplementary Figure 17. The crystal structures and docking model structures of TleC and MpnD. (**a**) The crystal structure of the TleC-**1**-DMSPP ternary complex. (**b**) The model structure of TleC comprexed with **1** and GPP. (**c**) The crystal structure of the MpnD-**1**-DMSPP ternary complex. (**d**) The model structure of MpnD complexed with **1** and GPP. The substrates (**1** and DMSPP) in TleC and MpnD are depicted by a blue and orange stick models, respectively. The amino acid residues located in the diphosphate binding site and the binding site of **1** are represented by limegreen and green stick models in TleC and salmon and magenta stick models in MpnD, respectively. Dashed yellow lines represent the distances between two atoms.

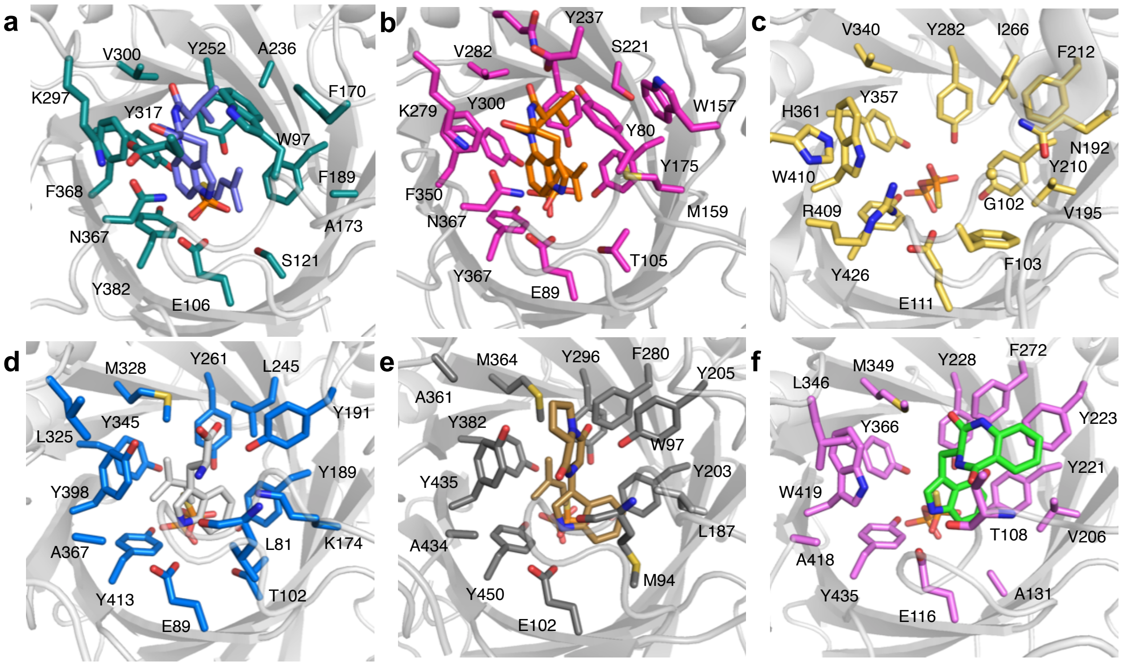

Supplementary Figure 18. Comparison of the active sites of TleC, MpnD, and other indole PTs. Comparison of the active sites of (a) TleC (green), (b) MpnD (magenta), (c) AnaPT from *N. fischeri* (yellow), (d) FgaPT2 from *A. fumigatus* (blue), (e) FtmPT1 from *A. fumigatus* (gray), and (f) CdpNPT from *A. fumigatus* (pink). The respective substrates **1**, L-tryptophan, brevianamide F, and (S)-benzodiazepinedione, for TleC, MpnD, FgaPT2, FtmPT1, and CdpNPT are represented by blue, orange, white, brown, and green stick models.

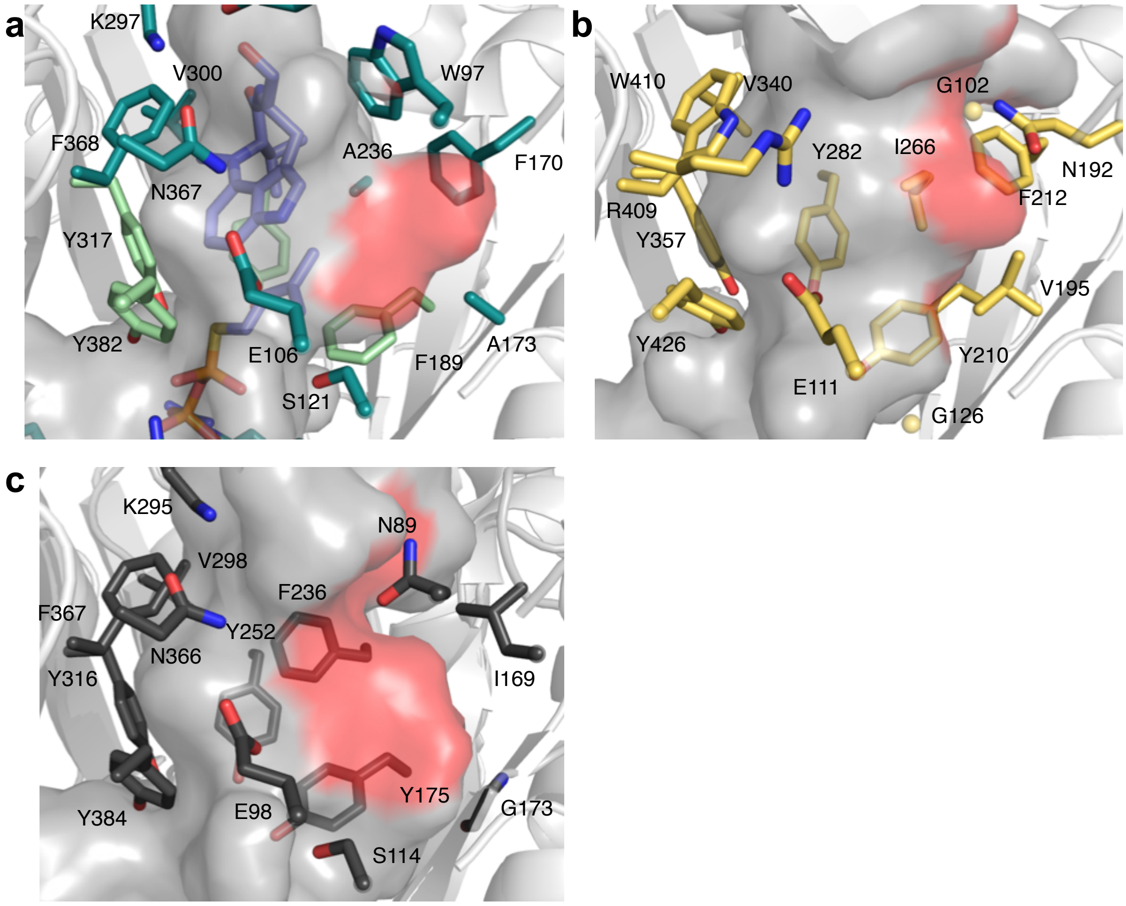

Supplementary Figure 19. Comparison of the active site cavity of crystal structure of TleC, AnaPT, and model structure of LtxC. Comparison of the active site cavity of (**a**) TleC (green), (**b**) AnaPT (yellow), and (**c**) model of LtxC (black). GPP binding pocket in each active site are highlighted by red surface.

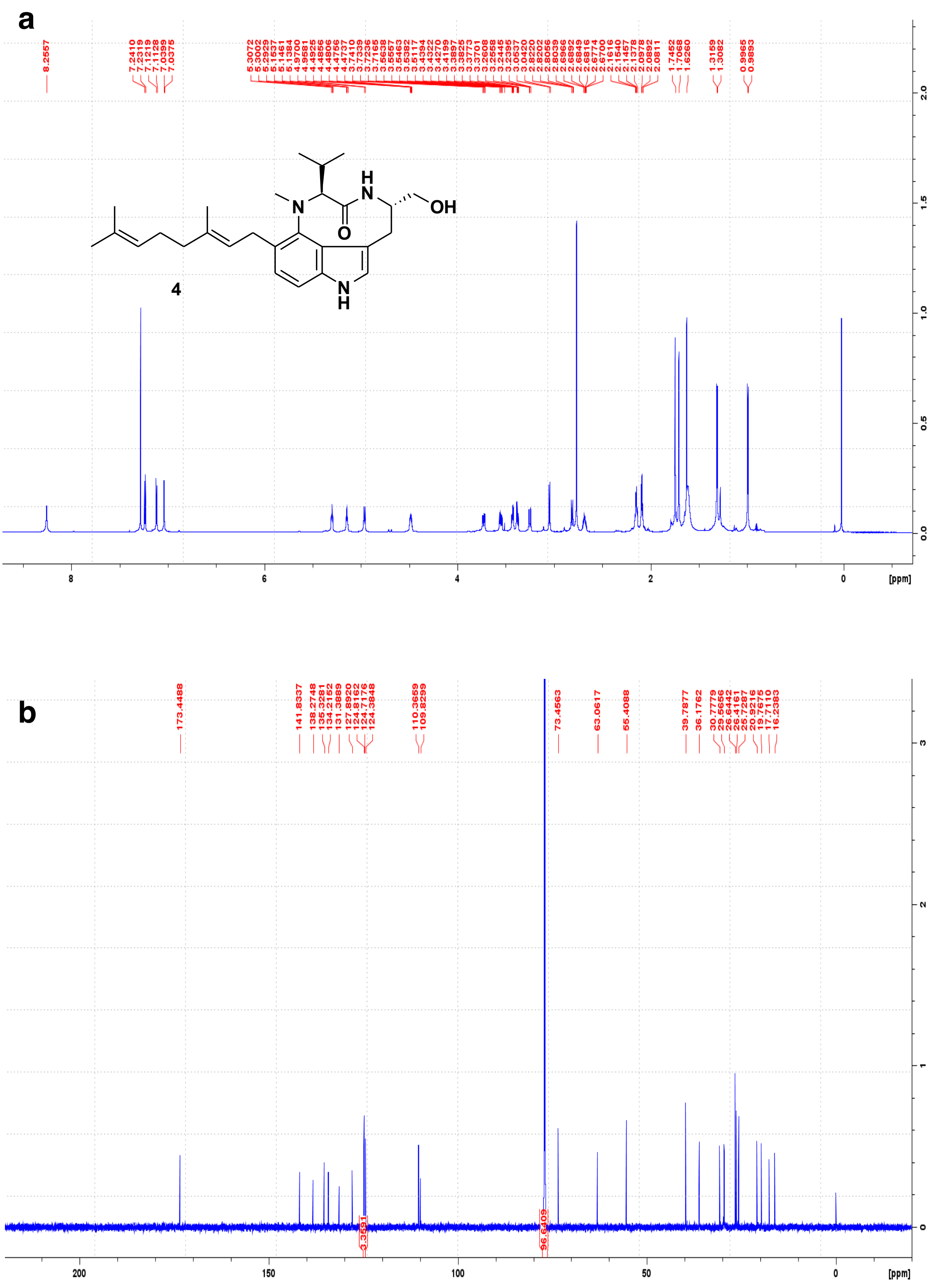
a
4
OH
[ppm]
b
[ppm]

c

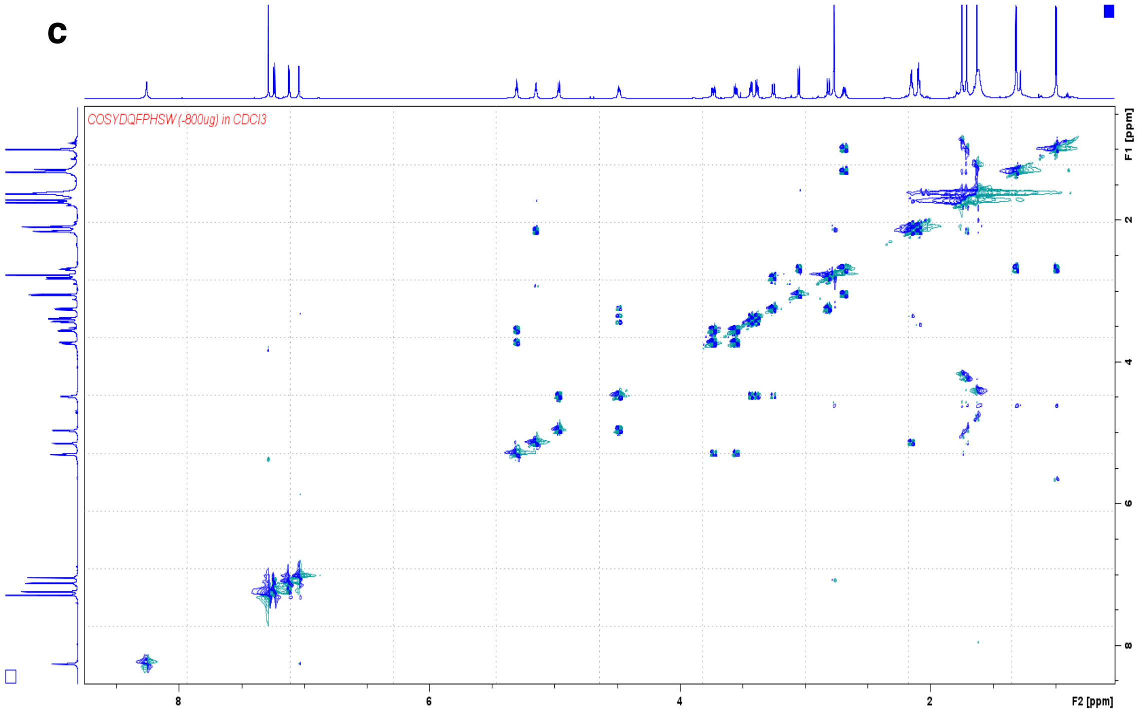
COSYDQFPHSW (-800ug) in CDCl3
F1 [ppm]
F2 [ppm]
2
4
6
8

d

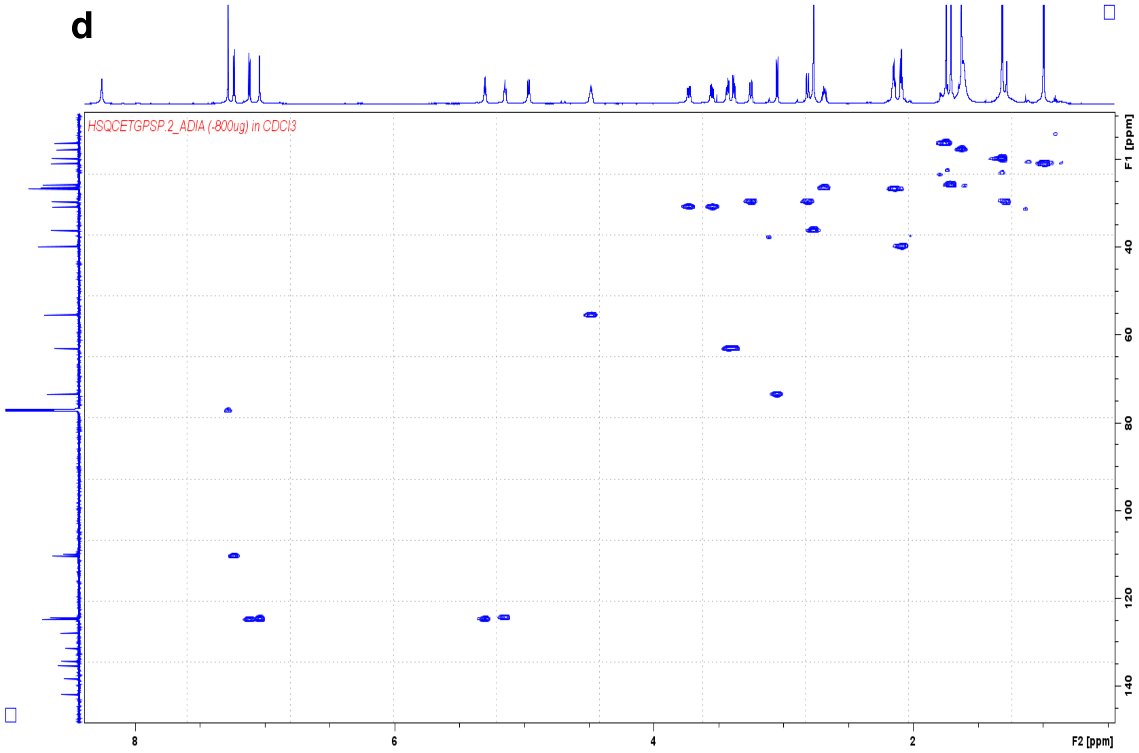
HSQCETGPSP.2_ADIA (-800ug) in CDCl3
F1 [ppm]
F2 [ppm]
40
60
80
100
120
140
8
6
4
2

e

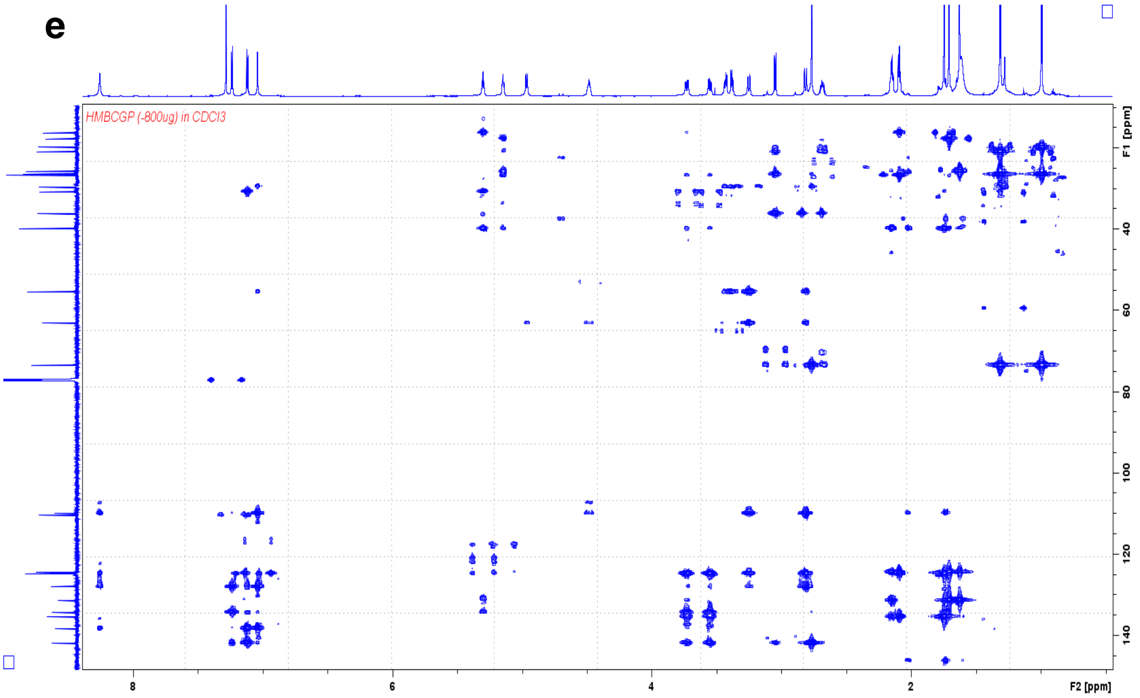

Supplementary Figure 20. NMR spectra of compound 4. (**a**) ^{1}H-NMR, (**b**) ^{13}C-NMR, (**c**) DQF-COSY, (**d**) HSQC, and (**e**) HMBC spectrum

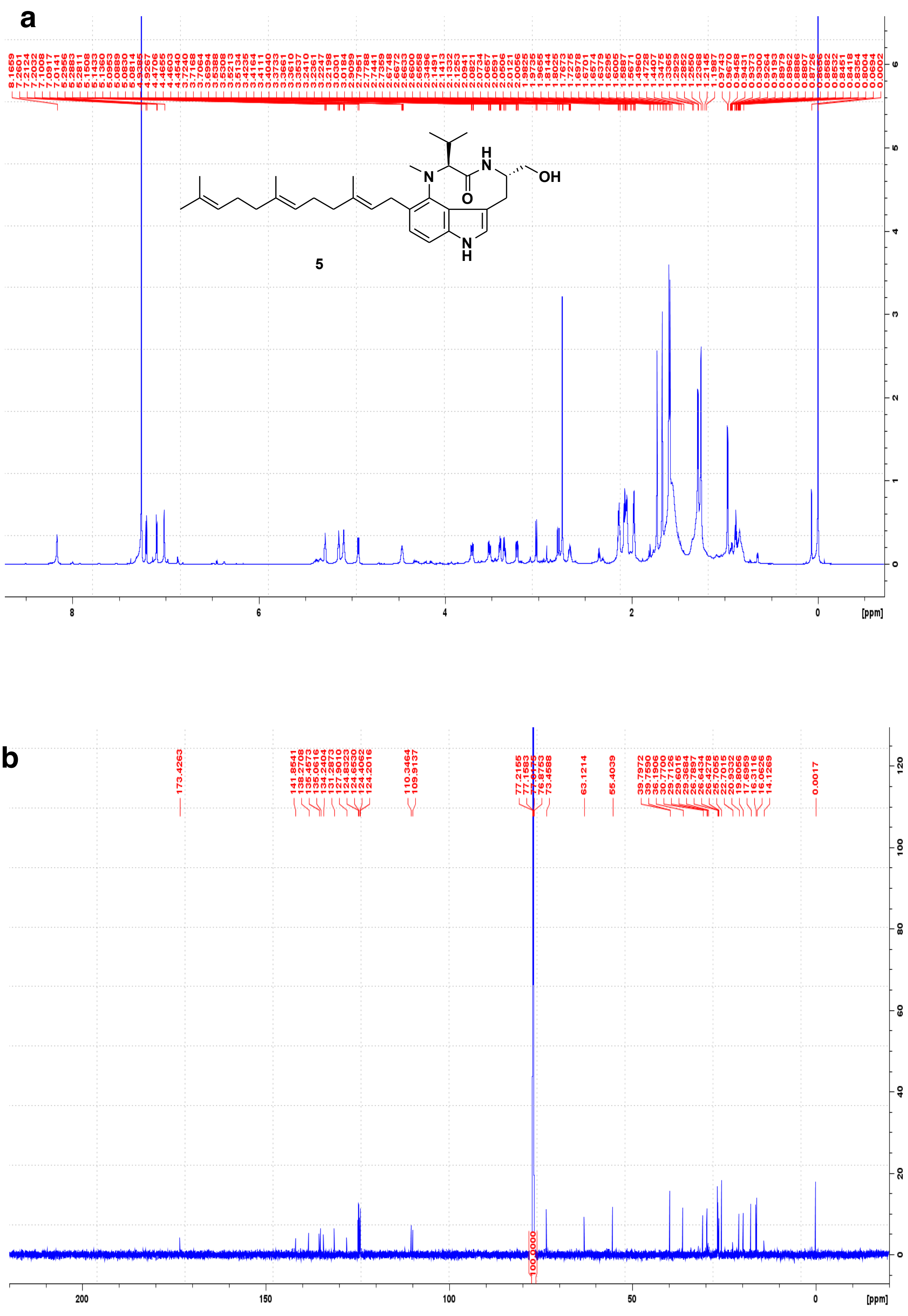

a
b
5
OH
[ppm]

c

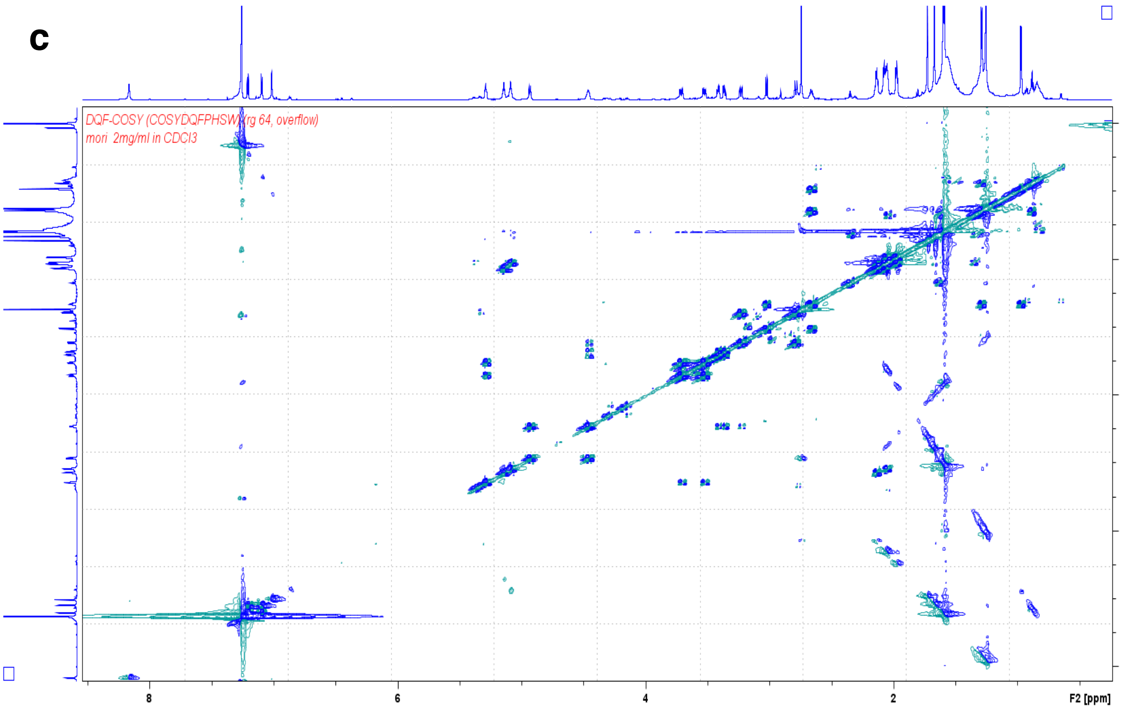
DQF-COSY (COSYDQFPHSW, rg 64, overflow)
mori 2mg/ml in CDCl3
F1 [ppm]
F2 [ppm]
8
6
4
2
8
6
4
2

d

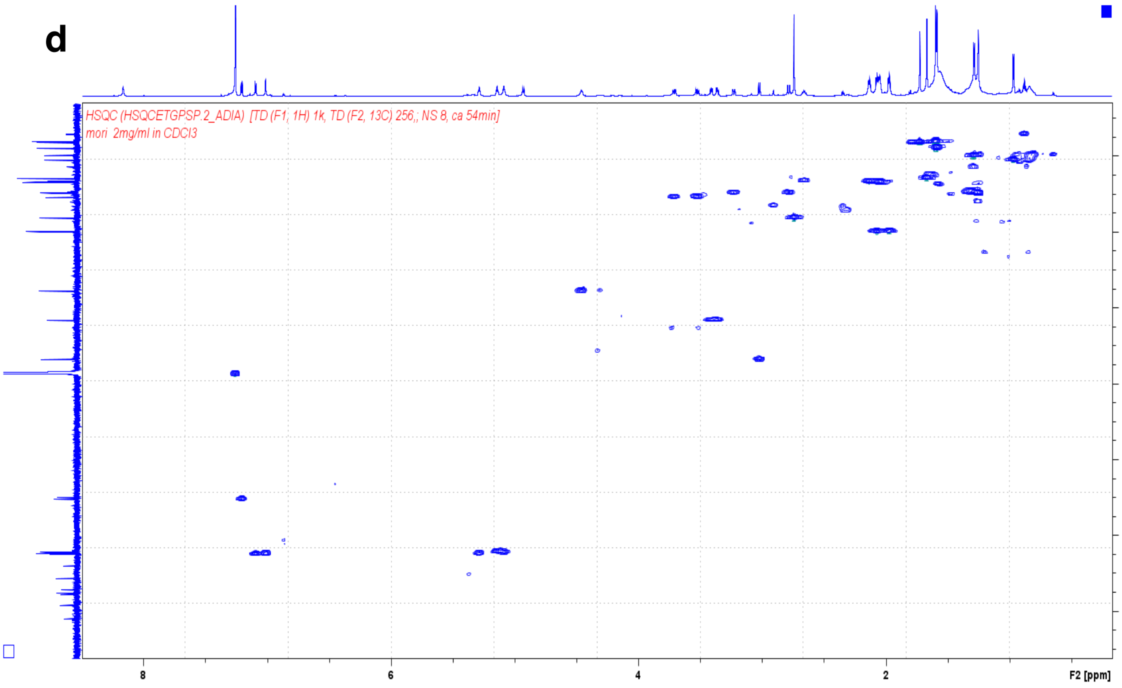
HSQC (HSQCETGPSP.2_ADIA) [TD (F1, 1H) 1k, TD (F2, 13C) 256,; NS 8, ca 54min]
mori 2mg/ml in CDCl3
F1 [ppm]
F2 [ppm]
8
6
4
2
20
40
60
80
100
120
140

e

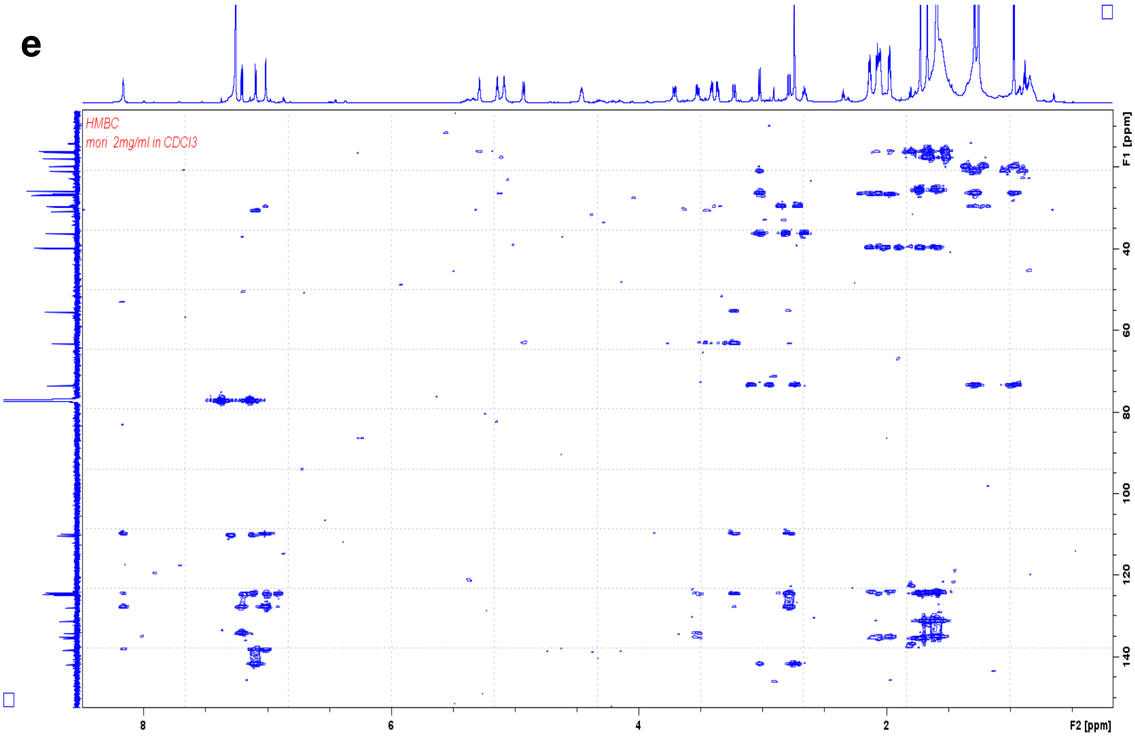

Supplementary Figure 21. NMR spectra of compound 5. (**a**) ¹H-NMR, (**b**) ¹³C-NMR, (**c**) DQF-COSY, (**d**) HSQC, and (**e**) HMBC spectrum
